# The Effect of Syringic Acid and Phenoxy Herbicide 4-chloro-2-methylphenoxyacetic acid (MCPA) on Soil, Rhizosphere, and Plant Endosphere Microbiome

**DOI:** 10.3389/fpls.2022.882228

**Published:** 2022-05-31

**Authors:** Elżbieta Mierzejewska, Magdalena Urbaniak, Katarzyna Zagibajło, Jaco Vangronsveld, Sofie Thijs

**Affiliations:** ^1^UNESCO Chair on Ecohydrology and Applied Ecology, Faculty of Biology and Environmental Protection, University of Łódź, Łódź, Poland; ^2^Environmental Biology, Centre for Environmental Sciences, Hasselt University, Hasselt, Belgium; ^3^Food Safety Laboratory, Research Institute of Horticulture, Skierniewice, Poland; ^4^Department of Plant Physiology and Biophysics, Institute of Biological Sciences, Faculty of Biology and Biotechnology, Maria Curie-Skłodowska University, Lublin, Poland

**Keywords:** 16S rRNA gene amplicons, 4-chloro-2-methylphenoxyacetic acid, zucchini, microbiome, syringic acid

## Abstract

The integration of phytoremediation and biostimulation can improve pollutant removal from the environment. Plant secondary metabolites (PSMs), which are structurally related to xenobiotics, can stimulate the presence of microbial community members, exhibiting specialized functions toward detoxifying, and thus mitigating soil toxicity. In this study, we evaluated the effects of enrichment of 4-chloro-2-methylphenoxyacetic acid (MCPA) contaminated soil (unplanted and zucchini-planted) with syringic acid (SA) on the bacterial community structure in soil, the rhizosphere, and zucchini endosphere. Additionally, we measured the concentration of MCPA in soil and fresh biomass of zucchini. The diversity of bacterial communities differed significantly between the studied compartments (i.e., unplanted soil, rhizospheric soil, and plant endosphere: roots or leaves) and between used treatments (MCPA or/and SA application). The highest diversity indices were observed for unplanted soil and rhizosphere. Although the lowest diversity was observed among leaf endophytes, this community was significantly affected by MCPA or SA: the compounds applied separately favored the growth of *Actinobacteria* (especially *Pseudarthrobacter*), while their simultaneous addition promoted the growth of *Firmicutes* (especially *Psychrobacillus*). The application of MCPA + SA together lead also to enhanced growth of *Pseudomonas*, *Burkholderia*, *Sphingomonas*, and *Pandoraea* in the rhizosphere, while SA increased the occurrence of *Pseudomonas* in leaves. In addition, SA appeared to have a positive influence on the degradative potential of the bacterial communities against MCPA: its addition, followed by zucchini planting, significantly increased the removal of the herbicide (50%) from the soil without affecting, neither positively nor negatively, the plant growth.

## Introduction

The presence of plant secondary metabolites (PSMs) such as flavonoids, coumarins, phenolic compounds, and terpenes, is known to modify the chemical and physical properties of soils. PSMs also serve as allelochemicals, protect plants against pathogens, act as substrates, and can induce pollutant catabolic pathways of soil microorganisms (Pilon-Smits, 2005; Singer, 2006; Zhou and Wu, 2012; Pino et al., 2016). They also shape the structure and function of plant-associated bacterial communities, i.e., rhizobacteria and endophytes, favoring the growth of suitable pollutant consumers (Uhlik et al., 2013; Qin et al., 2014; Eevers et al., 2016), which directly use their degradative capabilities to metabolize a given pollutant (Glick, 2010; Pawlik et al., 2015; Sauvêtre and Schroder, 2015; Khare et al., 2018; Wu et al., 2018). Thus, the role of PSMs in the degradation of pollutants can be substantial.

Due to their structural similarity, PSMs may have a considerable influence on the removal of structurally related pollutants (Uhlik et al., 2013; Hu et al., 2014). PSMs typically provide the energy for microorganisms to perform cometabolism, while the structurally similar pollutant is degraded as a secondary substrate (Musilova et al., 2016). Common examples of cometabolites are biphenyl and PCBs (Singer et al., 2003). However, PSMs can also be used as a primary source of carbon and energy by bacterial communities to support their growth and stimulate the expression of desirable genes involved in the catabolism of structurally similar pollutant. The degree of similarity between a PSM and a pollutant has been found to influence the rate of pollutant removal (Mierzejewska et al., 2019; Urbaniak et al., 2019a).

Earlier studies described syringic acid (SA) as a characteristic PSM for cucurbits (Blum et al., 2000; Campos et al., 2009; Kruczek et al., 2015; Shi et al., 2016), which are themselves known as effective phytoremediators of organic pollutants (Eevers et al., 2018; Urbaniak et al., 2019b,^2020^). The addition of SA to bacterial cultures not only enhanced MCPA removal but more importantly increased the number of detected functional genes responsible for the initiation of phenoxy herbicide biodegradation (Mierzejewska et al., 2019; Urbaniak et al., 2019a). Additionally, SA application and zucchini cultivation was found to decrease the toxicity of MCPA-contaminated soil (Mierzejewska et al., 2022).

4-chloro-2-methylphenoxyacetic acid (MCPA) is one of the most commonly used herbicides in Europe, and it is particularly persistent under the low winter temperatures, low soil organic carbon content, and acidic pH that are typical for Europe (Paszko et al., 2016). In areas with high contamination levels, heavy rainstorms facilitate the transport of MCPA residues from soil to water, resulting in the contamination of aquatic ecosystems (Matamoros et al., 2012; Rheinheimer dos Santos et al., 2020) to levels exceeding permissible threshold concentrations (Ignatowicz and Struk-Sokołowska, 2004; Rippy et al., 2017). These are believed to account for the adverse effects on living organisms reported in several worldwide studies (Pereira and Cerejeira, 2000; Nielsen and Dahllof, 2007; Palma et al., 2018; Morton et al., 2020).

Despite the large body of research performed on the effects of MCPA on soil- and water-inhabiting organisms, little is known about its influence on soil and plant-associated bacterial communities. Also, little attention has been devoted to the role of PSMs in the remediation of MCPA-contaminated soil. While our prior research has demonstrated that SA treatment enhanced MCPA transformation (Mierzejewska et al., 2019; Urbaniak et al., 2019a), no studies have examined the effects of such treatment on the structure of plant-associated bacterial communities (rhizobacteria and endophytes).

Consequently, this study aimed to determine whether adding SA, which is structurally like the herbicide, to unplanted and zucchini-planted soil contaminated with MCPA shapes the bacterial communities within the unplanted soil, rhizosphere, and plant endosphere (i.e., endophytic communities in roots and leaves). The obtained results could be applied to develop nature-based solutions for the removal of MCPA residues and to prevent their dispersal in the environment.

## Materials and Methods

### Materials

#### Soil

The potting soil was obtained from a certified supplier: Substral OSMOCOTE. Its composition was described as universal soil for plant growth, containing a mixture of peat, fertilizer, expanded clay, and silica and with the following properties: pH 4.3; 34.3% C, 2.2% N; 1.5 g P kg^–1^ soil, 2.5 g K kg^–1^ soil. This soil was selected based on previous experiments with cucurbits (Mierzejewska et al., 2022).

#### Plants

Based on previous investigations on the uptake of toxic organic pollutants by the cucurbits (Urbaniak et al., 2016; Wyrwicka et al., 2019; Mierzejewska et al., 2022), *C. pepo* L. “Atena Polka” (zucchini) was chosen for this study. The seeds were purchased from a certified supplier of garden seeds (W. Legutko) and were germinated under stable conditions in perlite for 5 days and grown in unamended potting soil for 7 days to select seedlings at the same growth stage.

#### Herbicide and Plant Secondary Metabolite

MCPA, C_9_H_9_ClO_3_ (≥95.0% purity, molecular weight 200.61 g mol^–1^, 0.825 g L^–1^ water solubility at 20°C, pKa value 3.07) was obtained from Sigma-Aldrich. The PSM used in this study was syringic acid (SA), C_9_H_10_O_5_ (≥95.0% purity, molecular weight 198.17 g mol^–1^, 5.78 mg ml^–1^ water solubility at 25°C, pKa value 4.34), also obtained from Sigma-Aldrich. The concentrations of MCPA (0.05 mM) and SA (0.125 mM) were selected based on earlier studies (Urbaniak et al., 2019a; Mierzejewska et al., 2022). Stock solutions of MCPA and SA were prepared in methanol.

### Methods

#### Experimental Setup

Each treatment was prepared in six replicates, with one seedling (zucchini) per pot, resulting in a total of six plants for each treatment variant. Unplanted soil variants were also included as an unplanted reference (Table 1). Soil humidity was adjusted to 60% v/w, and MCPA and SA were applied to the potting soil. The soil variants were kept for 1 h under the fume hood to allow the solvent to evaporate. Following this, the zucchini seedlings were planted into the potting soil.

**TABLE 1:** Abbreviations used for the different variants and their description.

Variants abbreviation	Compartment	MCPA	SA
Unplanted variants			
Us	Unplanted soil	−	−
Us + SA		−	+
Us + MCPA		+	−
Us + MCPA + SA		+	+
Planted variants			
ZuRh	Rhizosphere soil	−	−
ZuRh + SA		−	+
ZuRh + MCPA		+	−
ZuRh + MCPA + SA		+	+
ZuRo	Root endosphere	−	−
ZuRo + SA		−	+
ZuRo + MCPA		+	−
ZuRo + MCPA + SA		+	+
ZuLe	Leaves endosphere	−	−
ZuLe + SA		−	+
ZuLe + MCPA		+	−
ZuLe + MCPA + SA		+	+

*“+” means treatment; “−” means no treatment.*

All variants were cultivated in 500 cm^3^ soil pots, in a growth chamber at 23 ± 0.5°C based on a 16 h light/8 h dark cycle, with a photon flux density of 250 μmol m^–2^ s^–1^ during the light period, and 60% w/v soil humidity (Wyrwicka and Urbaniak, 2016). All variants were watered daily. The experiments were running for 20 days. Samples of unplanted soil and rhizosphere (rhizospheric soil and roots) soil were collected and stored at 4°C for further analyses. The aboveground biomass of the plants (stems and leaves) was determined subsequently. The leaves were collected and stored at 4°C for further analyses.

#### MCPA Concentration in Soil

The MCPA concentrations were determined in all Soil variants (unplanted and planted) at the beginning of the experiment and after 20 days (Us + MCPA; Us + MCPA + SA; Zu + MCPA; Zu + MCPA + SA). MCPA was determined according to the sample preparation method published by the European Commission (EURL-SRM, 2015, 2020). Briefly, 5.00 g ± 0.05 g of soil was weighed in a 50 ml Teflon centrifuge tube, and 10 ml of deionized water was added. Then, a 10 ml sample extraction solvent (1% formic acid in acetonitrile) was added. After shaking this mixture for 15 min using the QuEChERS Hand Motion Shaker (Eberbach model EL 680.Q.25 QuEChERS) with 450 osc., 4 g of magnesium sulfate and 1 g of sodium chloride were added, followed by shaking for 1 min. The suspension was centrifuged for phase Separation at 8,100 rpm for 5 min. The extract was then diluted (200 μl of extract with 700 μl of water, 50 μl of acetonitrile, and 50 μl of internal standard MCPA D6). This mixture was vortexed and filtered through a 0.22 μm PTFE directly into an amber HPLC vial.

The MCPA concentrations in the extracts were determined by highly selective liquid chromatography coupled with tandem mass spectrometry (LC-MS/MS—Agilent 1260 HPLC + 6460 Triple Quad LC/MS) (Agilent Technologies). The chromatography and mass spectrometry conditions are presented in Supplementary Tables 1, 2. The suitability of the method for analyzing MCPA residues in soil has been confirmed: the selectivity/specificity, limit of detection and quantification, linearity, precision, recovery, and expanded uncertainty of the method were validated. The obtained validation parameters met the criteria described in the document SANTE/12682/2019 (European Committee for Standardization, 2018).

#### Molecular Analysis: Microbial Community Structure Characterization

##### Rhizospheric Soil and Root Separation

The rhizospheric soil and roots were separated in 15 ml Falcon tubes containing 2.5 ml sterile PBS-S buffer amended with Tween 0.2 ml L^–1^ to separate rhizospheric soil tightly attached to roots. Subsequently, the rhizospheric soil was shaken off on the platform for 20 min at 180 rpm. The roots were then transferred to Falcon tubes containing sterile 2.5 ml of PBS-S buffer, rinsed, and subjected to surface sterilization. Rhizospheric soil was centrifuged for 20 min at 3,500 rpm, the supernatant was discarded and the resulting pellet was defined as a rhizosphere compartment (Bulgarelli et al., 2012).

##### Roots and Leaves Surface Sterilization

To isolate the DNA of endophytic bacterial communities, the roots and leaves were subjected to surface sterilization. The tissues were first rinsed in sterile distilled water to wash off the bulk dust. Subsequently, tissues were incubated in 70% ethanol (1 min), NaOCl (1% roots, 2.5% leaves; 1 min), 70% ethanol (1 min) for surface sterilization. Afterward, the samples were rinsed three times in sterile dH_2_O. Finally, 100 μl of the third rinsing water was transferred to a Petri dish containing an undiluted 869 medium (Mergeay et al., 1985) to check for sterility.

##### DNA Extraction

Total DNA from bulk and rhizospheric soil was isolated according to the DNeasy PowerSoil Pro Kit (Qiagen, Venlo, Netherlands). The surface-sterilized plant tissues (roots and leaves) were lyophilized before DNA extraction and snap-frozen in liquid nitrogen. Endophytic DNA was isolated according to PowerPlant^®^ Pro DNA Isolation Kit. DNA samples were quality checked using Nanodrop 2000 (Thermo Fisher Scientific, Wilmington, DE, United States) and stored at −20°C.

##### 16S rRNA Amplicon Sequencing of the Soil, Roots, and Leaves

The isolated DNA was used for the 16S rRNA gene amplification. DNA was isolated from unplanted variants, rhizospheric soil, surface-sterilized roots, and leaves.

###### Library Preparation and Illumina Sequencing

All DNA samples were subjected to bacterial 16S rRNA gene amplicon PCR. In the first round of 16S rRNA gene PCR, an amplicon of 291 bp was generated, using primers 515F-GTGYCAGCMGCCGCGGTAA and 806R- GGACTACNVGGGTWTCTAAT (Walters et al., 2016), with an Illumina adapter overhang nucleotide sequence, resulting in the following sequences, 515F-adapter: 5′-TCGTCGGCAGCGTCAGATGTGTATAAGAGACAG-3′ and 806R-adapter: 5′- GTCTCGTGGGCTCGGAGATGTGTATAAG AGACAG-3′. For the first round of PCR, the Q5 High-Fidelity DNA Polymerase system (M0491, NEB), a reaction volume of 25 μl per sample was prepared containing 1 μl of extracted DNA (final DNA-concentration per reaction 1–10 ng), 1x Q5 Reaction Buffer with 2 mM MgCl2, 200 μM dNTP mix, 1x Q5 High GC Enhancer (for the roots endophytes samples), 0.25 μM forward or reverse primer, and 0.02 U/μl Q5 High-Fidelity DNA polymerase, and for the plant endophytic samples (roots and leaves), additionally 2.5 μl mitoPNA blocker (PNA Bio PCR Blockers, 2021) (5 μM final concentration added from a 50 μM stock), 2.5 μl plastidPNA blocker (5 μM final concentration from 50 μM stock) (Kusstatscher et al., 2021) were used.

The PCR program started with an initial denaturation stage (3 min at 98°C), followed by a second denaturation (10 s at 98°C). Following this, 30 cycles were performed of the following three steps: for root and leaf endophytes, PNA clamping (10 s at 75°C), followed by annealing for V3V4 (30 s at 56°C) and extension (30 s at 72°C). The reaction was ended by a final 7 min extension at 72°C. The amplified DNA was purified using AMPure XP beads (Beckman Coulter) and a MagMax magnetic particle processor (ThermoFisher, Leuven, Belgium).

Subsequently, 5 μl of the cleaned PCR product was used for a second PCR attaching the Nextera indices (Nextera XT Index Kit v2 Set A; FC-131-2001, and D; FC-131-2004, Illumina, Belgium). For these PCR reactions, 5 μl of the purified PCR product was used in a 25 μl reaction volume and prepared following the 16S Metagenomic Sequencing Library Preparation Guide. The PCR conditions were the same as described above, but the number of cycles was reduced to 20, and a 55°C annealing temperature was used. The PCR products were cleaned with the Agencourt AMPure XP kit, and then quantified using the Qubit dsDNA HS assay kit (Invitrogen) and the Qubit 2.0 Fluorometer (Invitrogen).

Once the molarity of the sample was determined, the samples were diluted down to 4 nM using 10 mM Tris pH 8.5 before sequencing on the Illumina Miseq. The samples were sequenced using the Miseq Reagent Kit v3 (600 cycle) (MS-102-3003) and 15% PhiX Control v3 (FC-110-3001). For quality control, a DNA extraction blank and PCR blank were included throughout the process, and the ZymoBIOMICS Microbial Mock Community Standard (D6300) was used to test the efficiency of DNA extraction (Zymo Research).

###### Bioinformatic Processing of Reads

The sequences were demultiplexed using Illumina Miseq software; they were subsequently quality trimmed and the primers were removed using DADA2 1.10.1 (Callahan et al., 2016) in R version 3.5.1. The parameters for length trimming were set to keep the first 290 bases of the forward read and 200 bases of the reverse read, maxN = 0, MaxEE = (2,5) and PhiX removal. Error rates were inferred and the filtered reads were dereplicated and denoized using the DADA2 default parameters. After merging paired reads and removal of chimeras *via* the removeBimeraDenovo function, an amplicon sequence variant (ASV) table was built and taxonomy assigned using the SILVA v138 training set (Quast et al., 2012; Yilmaz et al., 2014). The resulting ASVs and taxonomy tables were combined with the metadata file into a Phyloseq object (Phyloseq, version 1.26.1) (McMurdie and Holmes, 2013). The pollutants were removed from the dataset using the Decontam package (version 1.2.1) applying the prevalence method with a 0.5 threshold value (Davis et al., 2018). A phylogenetic tree was constructed using a DECIPHER/Phangorn pipeline as described previously (Murali et al., 2018).

##### Data Visualization and Statistical Analyses

The ASV table was further processed by removing organelles (chloroplast, mitochondria), and prevalence was filtered using a 2% inclusion threshold (unsupervised filtering) as described by Callahan et al. (2016). The unfiltered data were subjected to alpha-diversity metrics, such as observed ASV count, Simpson’s and Shannon’s diversity index, using scripts from the MicrobiomeSeq package. Hypothesis testing was performed using analysis of variance (ANOVA) and Tukey’s HSD method using Statistica. For beta-diversity, the Bray–Curtis distances were calculated on unfiltered data using the *vegan* package (version 2.5.4), and the data were visualized using principal coordinate analysis (PCoA). Hypothesis testing was done by the Adonis function (*vegan*, version 2.5.5). To assess the homogeneity of variance, the Betadisper function (*vegan*, version 2.5.4) was used. Relative abundances were calculated and visualized in bar charts using Phyloseq. Differential abundance testing was done on unfiltered data using DESeq2 (version 1.22.2) (Bijnens et al., 2021). Hierarchical cluster analysis was performed to determine the differences between studied variants using PAST 4.0. All performed statistical tests were corrected for multiple testing, and *p* < 0.05 was considered statistically significant.

## Results

### MCPA Concentration in Soil

**FIGURE 1:**
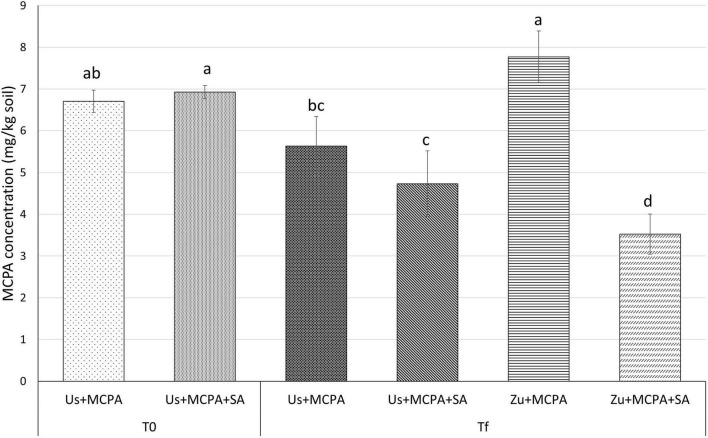
Concentrations of MCPA at the beginning of the experiment (T0) and after 20 days of incubation (Tf) in unplanted soil and in soil after zucchini cultivation. Variants: Us + MCPA, unplanted soil treated with MCPA; Us + MCPA + SA, unplanted soil treated with MCPA and SA; Zu + MCPA, soil after zucchini cultivation treated with MCPA; Zu + MCPA + SA, soil after zucchini cultivation treated with MCPA and SA. Different letters (a-d) indicate significant differences (Tukey’s HSD test, *p* < 0.05).

The MCPA content in the soils was determined at the beginning of the experiment (T0) and after 20 days (Tf) of incubation (Figure 1). The mean concentration of MCPA at T0 was 6.71 ± 0.270 mg g^–1^ in the soil amended with MCPA (Us + MCPA) and 6.93 ± 0.156 mg g^–1^ in soil amended with MCPA and SA (Us + MCPA + SA), respectively. After 20 days (Tf), the MCPA concentration in the Us + MCPA variant decreased to 5.63 ± 0.708 mg g^–1^ indicating about 18% reduction. In the soil amended with SA (Us + SA), the concentration of MCPA diminished to 4.73 ± 0.784 mg g^–1^, i.e., about 30% lower than at T0. The cultivation of zucchini in the MCPA-contaminated soil (Zu + MCPA) did not affect the MCPA concentration in the soil (7.77 ± 0.617 mg g^–1^). However, a substantial reduction of the MCPA concentration (∼50%) was observed in planted soil enriched with SA (Zu + MCPA + SA), where the final concentration of the herbicide was reduced to 3.53 mg g^–1^.

### Fresh Biomass

Plant fresh weight was significantly affected by the addition of MCPA (Table 2). Significantly higher aboveground biomasses were observed for the zucchini grown in soils without MCPA than those grown in soil amended with MCPA (Table 2). The same pattern was observed for fresh weights of leaves and stems measured separately, these values being significantly lower for variants amended with MCPA compared to unamended soil. The addition of SA alone had no significant effect on the plant’s fresh weight.

**TABLE 2:** Fresh weights of leaves and stems and total aboveground biomass of zucchini.

Variant*	Zu	Zu + SA	Zu + MCPA	Zu + MCPA + SA

Biomass (g)				
Leaves	27.11^a^ ± 0.7152	25.42^a^ ± 1.612	1.648^b^ ± 0.4830	1.400^b^ ± 0.1414
Stems	36.44^a^ ± 2.289	34.19^a^ ± 2.540	3.915^b^ ± 0.6303	4.040^b^ ± 0.1556
Total	63.54^a^ ± 2.694	59.61^a^ ± 1.346	5.563^b^ ± 0.7741	5.810^b^ ± 0.6744

Different letters (a-b) in the same rows indicate significant differences (Tukey’s HSD test, p ≤ 0.05). *Variants: Zu- zucchini from untreated soil; Zu+SA- zucchini from soil treated with SA; ZuRh+MCPA- zucchini from soil treated with MCPA; Zu+MCPA+SA- zucchini from soil treated with MCPA and SA.

### The Effect of Syringic Acid Application Without MCPA on Bacteria Community Structure

The changes in alpha diversity are presented in Table 3. Calculated indices (InvSimpson; Obs.ASVs; Shannon) were slightly lower in Us + SA than in Bs. The amendment of soil with SA increased the InvSimpson/Obs ASVs in the rhizosphere (ZuRh + SA) and roots endosphere (ZuRo + SA), and slightly increased the Shannon index in the roots endosphere (ZuRo + SA). Higher InvSimspon scores were observed in unamended ZuLe than in the SA-amended variant ZuLe + SA.

**TABLE 3:** Alpha-diversity analyses: three alpha-diversity indices [viz. InvSimpson: inverse of Simpson’s diversity index; obs.ASV: the observed amount of operational taxonomic units (ASV); H’–Shannon’s diversity index].

Indice	InvSimpson	Obs.ASV	H’

Variant*			
Us	101.6^a^	202.8^a^	4.875^a^
ZuRh	68.17^bef^	136.5^acg^	4.711^ad^
ZuRo	31.49^cg^	68.50^defg^	3.597^e^
ZuLe	3.527^cd^	7.667^de^	1.665^c^
Us + SA	91.30^ab^	173.3^ab^	4.690^ab^
ZuRh + SA	74.25^be^	164.3^ac^	4.440^ad^
ZuRo + SA	34.86^dfg^	94.33^bdegh^	3.842^de^
ZuLe + SA	2.731^c^	11.50^de^	1.692^c^
Us + MCPA	101.5^a^	200.0^a^	4.919^a^
ZuRh + MCPA	79.96^aef^	246.3^ah^	4.861^ad^
ZuRo + MCPA	39.01^dfg^	69.75^defg^	3.908^bde^
ZuLe + MCPA	2.415^c^	5.000^de^	1.491^c^
Us + MCPA + SA	92.15^ab^	223.7^a^	4.975^a^
ZuRh + MCPA + SA	69.52^be^	116.3^bc^	4.458^ad^
ZuRo + MCPA + SA	26.84^cg^	76.25^defg^	3.668^e^
ZuLe + MCPA + SA	3.647^c^	15.67^de^	1.559^c^

Different letters (a–h) in the same columns indicate significant results (Tukey’s HSD test, p ≤ 0.05). *Variants: Us, unplanted soil; ZuRh, zucchini rhizosphere soil; ZuRo, zucchini root; ZuLe, zucchini leaves; Us + SA, unplanted soil treated with SA; ZuRh + SA, zucchini rhizosphere soil treated with SA; ZuRo + SA, zucchini root from soil treated with SA; ZuLe + SA, zucchini leaves from soil treated with SA; Us + MCPA, unplanted soil treated with MCPA; ZuRh + MCPA, zucchini rhizosphere soil treated with MCPA; ZuRo + MCPA, zucchini root from soil treated with MCPA; ZuLe + MCPA, zucchini leaves from soil treated with MCPA; Us + MCPA + SA, unplanted soil treated with MCPA and SA; ZuRh + MCPA + SA, zucchini rhizosphere soil treated with MCPA and SA; ZuRo + MCPA + SA, zucchini root from soil treated with MCPA and SA; ZuLe + MCPA + SA, zucchini leaves from soil treated with MCPA and SA.

**FIGURE 2:**
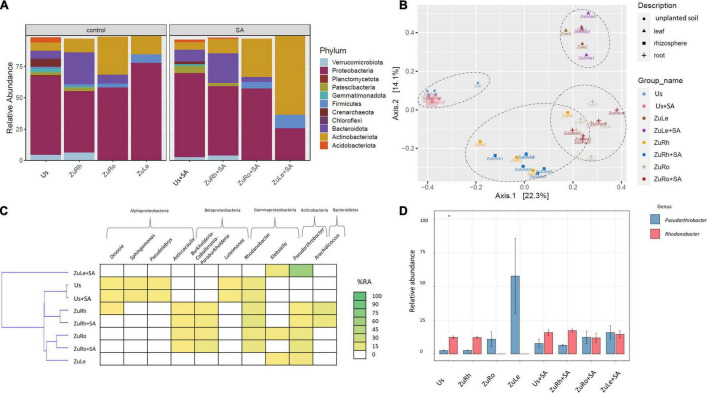
Bacterial communities structure based on 16S rRNA gene sequence for SA-amended variants in comparison to untreated variants: **(A)** relative abundances (%) of the bacterial community at the phylum level; **(B)** PcoA ordination plots of the results based on the Bray–Curtis distances of the classified 16S rRNA gene sequences; **(C)** Heat map of top 10 genera in each treatment; **(D)** Relative abundance (%) of Pseudarthrobacter and Rhodanobacter in the different treatments. Variants: Us, unplanted soil; ZuRh, zucchini rhizosphere soil; ZuRo, zucchini root; ZuLe, zucchini leaves; Us + SA, unplanted soil treated with SA; ZuRh + SA, zucchini rhizosphere soil treated with SA; ZuRo + SA, zucchini root from soil treated with SA; ZuLe + SA, zucchini leaves from soil treated with SA.

The addition of SA alone, without MCPA, influenced the composition of bacterial communities, both in soils and plant tissues (Figures 2A,B). A high abundance of *Proteobacteria* was observed in Us + SA (66.88%). However, the phyla *Acidobacteriota*, *Gemmatimonadota*, *Planctomycetoota*, *Firmicutes*, and *Chloroflexi* were not detected after the amendment of unplanted soil with SA (Us + SA). *Firmicutes* were also not detected in ZuRh + SA. After SA addition, the abundance of *Proteobacteria* in ZuLe + SA was three times lower (25.58%) than in ZuLe (77.81%), while the abundance of *Actinobacteria* was almost five times higher in ZuLe + SA (63.27%) than in ZuLe (14.67%).

The relative abundance of certain genera was also influenced by SA (Table 4 and Figures 2C,D). In contrast to the unamended variant (Bs), *Candidatus udaeobacter* was not detected in the Us + SA. *Devosia* and *Candidatus xiphinematobacter* genera were not detected in the ZuRh + SA. In turn, the relative abundance of *Mucilaginibacter* was six times higher in ZuRh + SA (6.588%) than in ZuRh (1.000%). SA had significant effects on the presence of several genera, especially in the roots endosphere: the relative abundance of *Dyella* was three times higher in ZuRo + SA (3.966%) than in ZuRo (1.000%). Although the presence of the genera *Ralstonia*, *Rhizobium*, *Massilia*, *Luteimonas*, *Klebsiella*, *Streptomyces*, and *Mucilaginibacter* was confirmed in ZuRo, they were not detected in ZuRo + SA. SA exerted a particularly strong effect on the dominant genus in leaves endosphere (Figure 2D), where the abundance of *Pseudarthrobacter* was significantly higher (61.50%) than in other variants. SA lowered the prevalence of *Paenibacillus* in ZuLe + SA five times (1.000%) in comparison to ZuLe (5.786%). Also, the relative abundance of *Paeniglutamicibacter* was four times lower in ZuLe + SA (1%) than in ZuLe (4.284%). In turn, higher abundances of *Pseudomonas* (13.72%), Klebsiella (11.77%), *Exiguobacterium* (5.138%), and *Paenisporosarcina* (4.417%) were observed in leaves endosphere after SA treatment (ZuLe + SA).

**TABLE 4:** Presence (+) of bacterial genera with relative abundance > 1% in the different variants.

Variant*	*Alphaproteobactera*					*Betaproteobacteria*				*Gammaproteobacteria*							*Firmicutes*					*Actinobacteria*			*Bacteroidetes*		*Verrucomicrobia*	
							
	*Devosia*	*Rhizobium*	*Sphingomonas*	*Pseudolabrys*	*Asticcacaulis*	*Burkholderia*	*Ralstonia*	*Pandoraea*	*Methylophilus*	*Luteimonas*	*Massiia*	*Dyella*	*Rhodanobacter*	*Salmonella*	*Klebsiella*	*Pseudomonas*	*Psychrobacillus*	*Exiguobacterium*	*Paenibacillus*	*Solibacillus*	*Paenisporosarcina*	*Streptomyces*	*Paeniglutamicibacter*	*Pseudarthrobacter*	*Arachidicoccus*	*Mucilaginibacter*	*Candidatus* *Xiphinematobacter*	*Candidatus* *Udaeobacter*
Us	+		+	+						+			+															+
ZuRh	+				+	+							+											+	+		+	
ZuRo		+			+	+	+				+		+		+							+		+		+		
ZuLe															+				+				+	+				
Us + SA	+		+	+						+			+															
ZuRh + SA					+	+							+											+	+	+		
ZuRo + SA					+	+						+	+											+				
ZuLe + SA															+	+		+			+			+				
Us + MCPA	+		+	+						+			+															
ZuRh + MCPA	+								+				+											+	+			
ZuRo + MCPA					+	+					+	+	+	+	+							+		+				
ZuLe + MCPA																			+					+				
Us + MCPA + SA	+		+							+			+															
ZuRh + MCPA + SA						+			+				+			+												
ZuRo + MCPA + SA			+		+	+		+			+	+	+	+	+									+				
ZuLe + MCPA + SA				+												+	+		+	+				+				

*Variants: Us, unplanted soil; ZuRh, zucchini rhizosphere soil; ZuRo, zucchini root; ZuLe, zucchini leaves; Us + SA, unplanted soil treated with SA; ZuRh + SA, zucchini rhizosphere soil treated with SA; ZuRo + SA, zucchini root from soil treated with SA; ZuLe + SA, zucchini leaves from soil treated with SA; Us + MCPA, unplanted soil treated with MCPA; ZuRh + MCPA, zucchini rhizosphere soil treated with MCPA; ZuRo + MCPA, zucchini root from soil treated with MCPA; ZuLe + MCPA, zucchini leaves from soil treated with MCPA; Us + MCPA + SA, unplanted soil treated with MCPA and SA; ZuRh + MCPA + SA, zucchini rhizosphere soil treated with MCPA and SA; ZuRo + MCPA + SA, zucchini root from soil treated with MCPA and SA; ZuLe + MCPA + SA, zucchini leaves from soil treated with MCPA and SA.

### The Effects of MCPA Application on the Bacterial Community Structure

MCPA had little effect on the alpha diversity in unplanted soil, rhizosphere, endosphere of roots, and leaves (Table 3). Additionally, in roots endosphere, MCPA enhanced the InvSimpson and Shannon indices. However, in the leaf, MCPA treatment lowered the InvSimpson value compared to the unamended variant.

**FIGURE 3:**
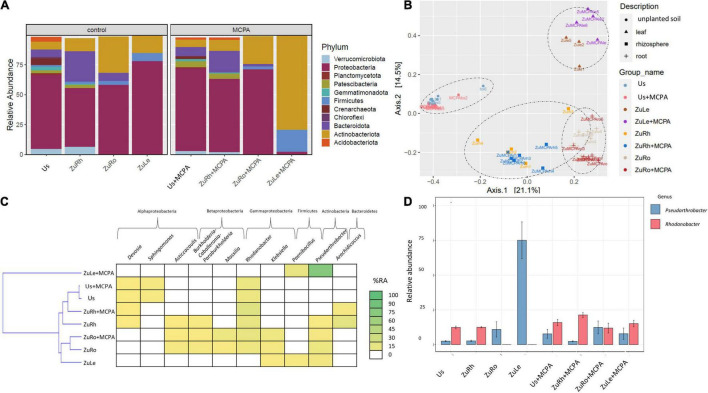
Bacterial community structure based on 16S rRNA gene sequence for MCPA-amended variants in comparison to untreated variants: **(A)** Relative abundances (%) of the bacterial community at the phylum level; **(B)** PcoA ordination plots of the results based on the Bray–Curtis distances of the classified 16S rRNA gene sequences; **(C)** Heat map of top 10 genera in each treatment; **(D)** Relative abundance (%) of *Pseudarthrobacter* and *Rhodanobacter* in the different treatments. Variants: Us, unplanted soil; ZuRh, zucchini rhizosphere soil; ZuRo, zucchini root; ZuLe, zucchini leaves; Us + MCPA, unplanted soil treated with MCPA; ZuRh + MCPA, zucchini rhizosphere soil treated with MCPA; ZuRo + MCPA, zucchini root from soil treated with MCPA; ZuLe + MCPA, zucchini leaves from soil treated with MCPA.

MCPA caused similar changes in the structure of the bacterial communities as SA (Figures 3A,B). The most abundant phylum in Us + MCPA was *Proteobacteria* (69.26%). In Us + MCPA, the relative abundance of *Acidobacteriota* (1.695%), *Crenarchaeota* (2.366%), and *Verrumicrobiota* (2.851%) was approximately two times lower than in Us (being: 4.124, 5.563, and 2.851%, respectively). In contrast, the abundance of *Patescibacteria* was two times higher in Us + SA (4.644%) than in Us (2.256%). *Proteobacteria* also predominated the bacterial community in ZuRh + MCPA (61.20%). In contrast, the relative abundance of *Actinobacteria* in ZuRh + MCPA increased by a factor of two (2.135%) in comparison to ZuRh (1.000%). A three times lower abundance of *Verrumicrobiota* was observed for ZuRh + MCPA (1.966%) than in ZuRh (6.469%). The relative abundance of *Actinobacteria* and *Firmicutes* in ZuLe + MCPA increased after MCPA treatment (78.90 and 18.41%, respectively), in comparison to ZuLe (14.67 and 7.10%, respectively). In contrast, the abundance of *Proteobacteria* from ZuLe + MCPA (77.81%) was approximately thirty times lower than in ZuLe (2.443%).

MCPA also changed the prevalence of individual genera (Table 4 and Figures 3C,D). The addition of MCPA to unplanted soil decreased the abundance of *Candidatus udaeobacter* by five times from 5.852% in Us + MCPA + SA to 1.000% in Us + MCPA. The genera *Asticcacaulis*, *Burkholderia*, and *Candidatus xiphinematobacter* were not detected in ZuRh + MCPA. In turn, the abundance of *Methylophilus* was six and half times higher in ZuRh + MCPA (6.510%) than in ZuRh (1.000%). Furthermore, higher values of the abundance of *Rhodanobacter* were observed for ZuRh + MCPA (20.52%) than in ZuRh (14.73%). In roots endosphere (ZuRo + MCPA), the abundances of *Dyella* and *Salmonella* (5.410 and 3.419%, respectively) were higher than in ZuRo (1.000 and 1.000%, respectively). In contrast, after the amendment of soil with MCPA *Mucilaginibacter*, *Ralstonia* and *Rhizobium* were not detected in roots endosphere (ZuRo + MCPA). MCPA had a particularly strong effect on the dominant genera in leaves endosphere (Figures 3C,D): the abundance of *Pseudarthrobacter* was almost eight times higher in ZuLe + MCPA (78.09%) than in ZuLe (10.12%). Furthermore, the genus *Paeniglutamicibacter* was not detected in ZuLe + MCPA.

### The Effects of Simultaneous Application of MCPA + SA on the Bacterial Community Structure

Statistically significant differences in alpha diversity were found between the studied compartments, particularly regarding the InvSimpson index (Table 3). For unplanted soil, the InvSimpson was slightly lower in Us + MCPA + SA than in Bs, and for roots endosphere, the index for ZuRo + MCPA + SA was lower than in ZuRo. However, in rhizosphere and leaves, the values were higher in the MCPA-treated variants than in untreated ones. In addition, a lower value of Obs. ASV was observed in ZuRh + MCPA + SA than in ZuRh.

**FIGURE 4:**
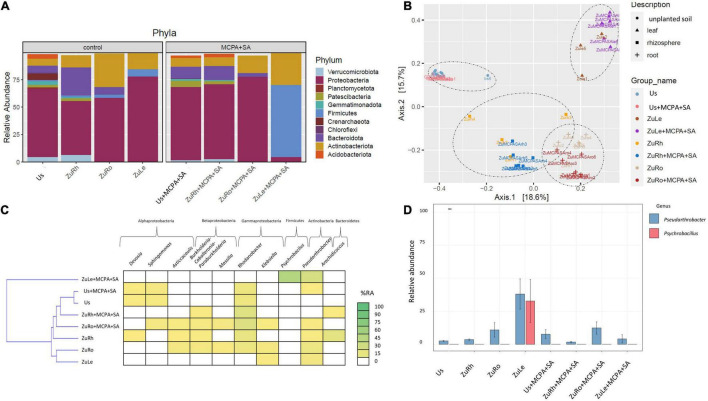
Bacterial community structure based on 16S rRNA gene sequence for MCPA + SA-amended variants in comparison to untreated variants: **(A)** Relative abundances (%) of the bacterial community at the phylum level; **(B)** PcoA ordination plots of the results based on the Bray–Curtis distances of the classified 16S rRNA gene sequences; **(C)** Heat map of top 10 genera in each treatment; **(D)** Relative abundance (%) of *Pseudarthrobacter* and *Psychrobacillus* in the different treatments. Variants: Us, unplanted soil; ZuRh, zucchini rhizosphere soil; ZuRo, zucchini root; ZuLe, zucchini leaves; Us + MCPA + SA, unplanted soil treated with MCPA and SA; ZuRh + MCPA + SA, zucchini rhizosphere soil treated with MCPA and SA; ZuRo + MCPA + SA, zucchini root from soil treated with MCPA and SA; ZuLe + MCPA + SA, zucchini leaves from soil treated with MCPA and SA.

The simultaneous addition of MCPA and SA to the soil affected both the soil and endophytic bacterial communities (Figures 4A–D). The predominant phylum in Us + MCPA + SA was *Proteobacteria* (69.26%). In turn, in US + MCPA + SA *Firmicutes*, *Planctomycetota*, and *Crenarchaeota* were not detected. *Proteobacteria* was also the predominant phylum in the rhizosphere ZuRh + MCPA (61.20%). The prevalence of *Bacteroidota* in ZuRh + MCPA + SA (12.32%) was two times higher in ZuRh (25.23%) than in ZuRh + MCPA + SA. Also, the relative abundance of *Proteobacteria* in roots endosphere (ZuRo + MCPA + SA) was higher (71.14%) than the abundance of other phyla. The prevalence of *Actinobacteria* was two times higher in ZuRo (30.23%) than in ZuRo + MCPA + SA (15.79%). The phylum *Firmicutes* was not detected in ZuRo + MCPA + SA, whereas its presence was confirmed in ZuRo (3.41%). In turn, *Firmicutes* dominated in ZuLe + MCPA + SA. The relative abundance of *Firmicutes* in ZuLe + MCPA + SA (65.39%) was almost seven times higher than in ZuLe (7.097%). The abundance of *Actinobacteria* was two times higher in ZuLe + MCPA + SA (29.68%) than in ZuLe (14.67%).

The addition of MCPA + SA to the soil had substantial effects on the presence of certain genera (Table 4 and Figures 4C,D). The genera *Pseudolabrys* and *Candidatus udaeobacter* were not detected in Us + MCPA + SA. In ZuRh + MCPA + SA, the highest relative abundance was observed for *Rhodanobacter* (21.23%) (Figure 4C). The abundance of *Methylophillus* was eight times higher in ZuRh + MCPA + SA (7.953%) than in ZuRh (1.000%). In turn, *Devosia*, *Asticcacaulis*, *Arachidococcus*, and *Candidatus xiphinematobacter* genera were not detected in ZuRh + MCPA + SA. Further, the highest observed relative abundance in ZuRo + MCPA + SA was noted for *Rhodanobacter* (23.56%). The amendment of soil with MCPA and SA enhanced the occurrence of *Sphingomonas* (7.485%) and *Pandoraea* (3.100%) in roots endosphere (ZuRo + MCPA + SA), whereas these genera were not detected in ZuRo. Neither *Ralstonia*, *Rhizobium*, *Streptomyces* nor *Mucilaginibacter* genera were detected in roots after MCPA and SA amendment (ZuRo + MCPA + SA). In ZuLe + MCPA + SA, the genus *Psychrobacillus* (48.28%) predominated the bacterial community, followed by *Pseudarthrobacter* (21.56%) (Figure 4D). In ZuLe + MCPA + SA, the genera *Pseudomonas* (3.380%) and *Solibacillus* (3.611%) were also detected, while *Klebsiella* and *Paeniglutamicibacter* were not observed.

### Bacterial Diversity in the Different Compartments

Considerable variations were observed in the alpha diversities of unplanted soil, rhizosphere soil, roots, and leaves endosphere (Table 3). The MANOVA analysis (Table 5) revealed that the diversity indices varied significantly in the function of the compartment (unplanted soil, rhizospheric soil, roots, and leaves endosphere). The highest diversity indices (Table 3) were observed in unplanted soil, ranging from 91.30 for Us + SA to 101.6 for Us (Inv. Simpson); from 173.3 for Us + SA up to 223.7 for Us + MCPA + SA (observed ASV); and from 4.690 for Us + SA up to 4.975 for Us + MCPA + SA (Shannon index).

**TABLE 5:** Main effects of categorical factors (compartment vs. SA vs. MCPA treatment) on diversity indices, estimated using Multivariate Tests of Significance (MANOVA).

Categorical factor	*F*	*p*
Compartment	55.91	0.0000*
SA	0.572	0.637
MCPA	0.508	0.680
Compartment*SA	1.21	0.304
Compartment*MCPA	0.472	0.889
SA*MCPA	0.716	0.550
Compartment*SA*MCPA	2.32	0.0226*

*Significant p < 0.05.

Slightly lower Inv. Simpson indices were observed in the rhizosphere, i.e., the highest value was found for ZuRh + MCPA (79.96) and the lowest for ZuRh (68.17). Lower diversity indices were observed in roots and leaves endosphere than in bulk and rhizospheric soil, ranging from 2.415 for ZuLe + MCPA to 39.01 for ZuRo + MCPA (Inv. Simpson), from 5.000 for ZuLe + MCPA to 94.33 for ZuRo + SA (observed ASV), and from 1.559 for ZuLe + MCPA + SA to 3.908 for ZuRo + MCPA (Shannon index).

Furthermore, the beta-diversity analysis indicated clustering (Figures 2B, 3B, 4B) of samples from different compartments. The MANOVA analysis demonstrated significant compartment specificity of the bacterial and fungal community structure (*p* < 0.001).

A compartment specificity was observed for the occurrence of specific genera irrespective of the treatment (Table 4). For example, the occurrence of *Devosia*, *Sphingomonas*, and *Luteimonas* was observed only in unplanted variants. Similarly, *Asticaccaulis* and *Burkholderia* were present in the roots endosphere of all variants. In turn, *Rhodanobacter* was observed in all variants of unplanted soil, rhizosphere, and roots endosphere, but not in the leaves endosphere.

## Discussion

Recent research has provided a comprehensive insight into the interactions between bacteria and plants in contaminated environments (Tardif et al., 2016; Thijs et al., 2018). It appears that the structure of bacterial communities in soils and plants is determined by the presence of pollutants. However, our knowledge about the effects of plant root exudates (including PSMs, such as SA) on the communities of bacteria in contaminated environments remains limited. Some studies have shown that certain phenolic compounds, such as benzoic acid, can enhance the biodegradative activity of bacteria in soil (Mandal et al., 2010; Zwetsloot et al., 2020). However, phenolic compounds can exert contrasting effects on microbial communities depending on the prevailing conditions (Zwetsloot et al., 2020).

While most studies have examined the biodegradation of phenoxy herbicides, especially 2,4-D (2,4-dichlorophenoxy acid) in soil, the most widely-used pesticide in European agriculture is MCPA. MCPA is usually sprayed as a commercial formulation in the form of amines, sodium salts, or esters to control perennial and broadleaf annual weeds (Paszko et al., 2016). Higher phenoxy herbicide (i.e., MCPA) concentrations are mostly detected in regions with ongoing intensive agriculture (Bianchi et al., 2017), although residuals of MCPA can be transported with intensive surface run-off and become a threat to non-target organisms. Monitoring studies showed that MCPA was present in 33–60% of the studied sites along the river tributaries in Poland (Zagibajło et al., 2017; Jarosiewicz et al., 2018). Thus, the development of methods for the prevention of MCPA residuals leaching is of the highest importance. Still, little attention has been paid to the potential of selected plants as phytoremediators of soils contaminated with phenoxy herbicides. The members of the *Cucurbitaceae* family are particularly promising candidates due to their ability to extract, translocate, and accumulate highly toxic persistent organic pollutants from soils (Hülster et al., 1994; White et al., 2003; Inui et al., 2008; Zhang et al., 2009; Wyrwicka et al., 2014; Urbaniak et al., 2016) and several cucurbit species, such as zucchini (*C. pepo* cv “Atena Polka”), have demonstrated resistance to MCPA (Mierzejewska et al., 2022).

Therefore, this study investigates the effects of a specific PSM (SA) and zucchini cultivation on the removal of MCPA from the soil. In unplanted MCPA-contaminated soil, amendment with SA led to a higher decrease of MCPA (30%) than in untreated soil (18%) (Figure 1). However, higher MCPA removal (50%) was observed in the planted condition amended with SA. Another study showed that the removal of 2,4-D from water, using the plant species *Plectranthus neochilus*, was 49% after 30 days (Ramborger et al., 2017). Furthermore, Germaine et al. (2006) demonstrated that endophyte-enhanced phytoremediation significantly improves the capacity for removal of 2,4-D. Our study showed that SA improves the removal of MCPA from unplanted soil. However, when SA application and zucchini cultivation are combined, the decrease of the herbicide concentration in the soil is significantly greater.

Although SA enhanced the removal of MCPA from soil (Figure 1), MCPA had a detrimental effect on fresh biomass (Table 2). Indeed, MCPA application was associated with 90% lower aboveground biomass in the planted variants. Phenoxy herbicides are transported to meristems, causing uncontrolled growth and consequently damaging the development of plant tissues (Grossmann, 2003). SA has been found to alleviate the toxic effects of MCPA (Mierzejewska et al., 2022), however, these studies investigated higher initial concentrations of both compounds. Kováčik et al. (2010) showed that certain phenolic acids, e.g., salicylic acid, can alleviate stress symptoms in plants. On the other hand, Zhou et al. (2014) suggested that SA can exhibit phytotoxic effects. Blum (1996) reported the concentration, composition, and synergism of individual phenolic compounds to be crucial for plant growth inhibition in the environment. Hence, we claim that despite improving MCPA-removal efficiency, the application of SA throughout the incubation time did not enhance the plant growth-promoting properties of the system.

The above-mentioned observations are as per earlier studies indicating that SA significantly enhanced the removal of MCPA from liquid cultures enriched with microorganisms derived from agricultural soil (Mierzejewska et al., 2019; Urbaniak et al., 2019a). Hence, this study also examines the effects of SA and MCPA, used individually or in combination, on the composition of bacterial communities within the soil, rhizosphere, and the zucchini plant itself.

The effects on the diversity of bacteria were found to be compartment-specific, with an interaction between SA and MCPA (Table 5). The highest diversity indices values were found for unplanted bulk and rhizospheric soil, and the lowest in leaves. Approximately, 10% lower values of diversity indices were observed after SA application (Table 3). Indeed, it has previously been reported that SA has antimicrobial activity against some bacterial strains (Shi et al., 2016). Our findings also indicate that the amendment of soil with MCPA did not affect diversity indices significantly (Table 3). Also, Ławniczak et al. (2016) found that the application of oligomeric herbicidal ionic liquids with MCPA did not cause any changes in the diversity indices. In variants amended with SA and MCPA diversity indices (i.e., Obs.ASV) were higher than in untreated variants. Also, Lipthay et al. (2004) reported higher species richness and Shannon index in herbicide-acclimated sediments. This effect can be explained by the direct and indirect effects of the applied compounds (MCPA and SA).

Formed metabolites and root exudates produced in response to stress conditions can also influence the structural diversity (Uhlik et al., 2013; Musilova et al., 2016). It is important to emphasize that interactions between the studied compartments and the application of both compounds, MCPA and SA, determined the diversity of bacteria.

After the addition of phenolic compounds (SA and MCPA), the bacterial communities in the unplanted soil, rhizospheric soil, and root endosphere were found to be dominated by *Proteobacteria* and differed only slightly from those of the untreated variant (Figures 2A, 3A, 4A). Also, the addition of DDE (0.0001 mg/L) to vermiculite led to the dominance of *Proteobacteria* in the roots endosphere of zucchini (Eevers et al., 2016). Similarly, Tejada et al. (2010) report that the use of MCPA (1.5 L/ha) did not appear to affect microbial structural diversity in soil. In contrast, it has been found that microbial communities isolated from the rhizosphere of soil contaminated with mecocrop (MCPP; 0.53 or 1.06 g/L) differed substantially from those isolated from unplanted soil (Lappin et al., 1985). The above-mentioned results show that in unplanted soil, rhizospheric soil, and roots endosphere, the amendment of soil with SA and MCPA not significantly affect the predominant taxa.

However, the application of MCPA and SA, either separately or together, considerably affected the composition of the endophytic bacterial community in leaves, although they demonstrated the lowest diversity indices (Figures 2–4). MCPA is absorbed through both leaves and roots and is translocated throughout the plant (*via* xylem and phloem) to the meristematic regions (Polit et al., 2014). The induced changes result in a series of biochemical and physiological processes which influence and disturb the morphology of plant roots, stems, and leaves (Grossmann, 2009). The observed changes in the composition of the plant endosphere microbiome can be due to the application of herbicides. Endophytes in plants can directly contribute to the detoxification of pollutants in plants or promote plant growth in stress conditions (Tétard-Jones and Edwards, 2016). However, the latter was not observed, since fresh weight was not higher after the simultaneous amendment of SA and MCPA. To the best of our knowledge, this is the first study showing that after the application of phenolic compounds (i.e., SA and MCPA) to soil, the most profound changes in structural diversity across the plant microbiome are observed in the leaves.

Further investigation of structural diversity showed that amendment of the soil with SA or MCPA led to the prevalence of the *Actinobacteria* (genus *Pseudarthrobacter*) in the leaves endosphere (Figures 2D, 3D). Similarly, Zhou et al. (2018) found that amendment of soil with the phenolic compound p-coumaric acid enhanced the relative abundances of *Actinobacteria* (i.e., *Pseudarthrobacter*) in the rhizosphere. *Actinobacteria* are described as common endophytic microorganisms, which can have multiple beneficial functions in plants, such as the production of antimicrobial agents (Musa et al., 2020) and biodegradation of petroleum and plastic compounds (Singh and Dubey, 2018). Combined MCPA and SA treatment, however, resulted in *Firmicutes* (genus *Psychrobacillus*) becoming the predominant taxon in leaves endosphere (Figure 4D). Regar et al. (2019) suggest that an increase in the abundance of *Firmicutes* can be a response to environmental stress such as herbicide contamination. Rangjaroen et al. (2019) mentioned that endophytic strains of *Bacillus* isolated from an herbicide-treated environment can enhance the resistance of plants to fungal pests. Our observations confirmed that the amendment of the soil with SA and MCPA favored the presence of *Actinobacteria* (genus *Pseudarthrobacter*) and *Firmicutes* (genus *Psychrobacillus*) in leaves.

The shifts in bacterial communities that occur after SA treatment could influence the degradative capacity against MCPA. Various genes belonging to the *tfd* cluster (responsible for initial steps of phenoxy herbicides biodegradation) have been identified in several taxa, e.g., *Burkolderia*, *Pseudomonas*, *Achromobacter*, *Delftia*, *Bradyrhizobium*, and *Cupriavidus* (Suwa et al., 1996; Kamagata et al., 1997; Baelum et al., 2010). Previous studies also demonstrated that SA doubled the presence of *tfdA* genes (Mierzejewska et al., 2019; Urbaniak et al., 2019a). In our study, the enrichment of soil with SA induced the occurrence of certain *Proteobacteria* genera such as *Pseudomonas* in leaves endosphere (Table 4), while the addition of MCPA + SA positively influenced the presence of *Pseudomonas* and *Burkholderia* in the rhizosphere (Table 4). Germaine et al. (2006) demonstrated that the endophytic strain *Pseudomonas putida* VM1450 enhanced the removal of 2,4-D from soil and demonstrated high biodegradative potential. In addition, the presence of *Burkholderia* in soils has been confirmed in our previous studies (Mierzejewska et al., 2019; Urbaniak et al., 2019a). Our findings indicate that combined MCPA + SA treatment induced the presence of *Sphingomonas* and *Pandoraea* in the roots endosphere (Table 4). Several *Sphingomonas* strains have been reported as being MCPA-degraders (Gözdereliler et al., 2013; Liu et al., 2013; Nielsen et al., 2013), and *Pandoraea* was found to substantially contribute to the degradation of lindane (HCH) in water and soil slurries (Okeke et al., 2002); however, to our knowledge, no studies have examined whether this genus was able to degrade phenoxy herbicides or related phenolic compounds. The above findings suggest that the amendment of the soil promoted the growth of specialized taxa; however, they were not the predominant taxa in the investigated compartments. Still, the functions of the identified strains need to be further confirmed by culturing techniques and molecular as well as metabolic analysis.

## Conclusion

The above findings show that the addition of SA can enhance the removal of MCPA from unplanted soil. This removal is further enhanced by combining SA amendment with the cultivation of zucchini; although SA itself did not have a positive effect on the zucchini growth. The application of both phenolic compounds (i.e., MCPA and SA) led to changes in bacterial diversity: the highest bacterial diversity indices were observed for unplanted soil and rhizospheric soil, and the lowest in leaves. Despite the lowest diversity, the leaf endophytes were strongly affected by the addition of the studied phenolic compounds favoring the growth of the phyla *Actinobacteria* (especially *Pseudarthrobacter* spp.) and *Firmicutes* (especially *Psychrobacillus*). SA also promoted the growth of specific genera in the rhizosphere (*Burkholderia*, *Pseudomonas*, *Sphingomonas*) and in leaves endosphere (*Pseudomonas*), which were previously reported to harbor functional genes for MCPA biodegradation. Hence, it appears that SA not only influenced the structure of the bacterial communities in either MCPA-contaminated soil and zucchini, but also enhanced the bacterial degradation of the herbicide in the soil–plant system.

This is the first study to collectively examine different aspects of MCPA removal from soil: biostimulation (use of the PSM as an enhancer of MCPA removal) and phytoremediation (use of zucchini as a potential tool for removal of the herbicide) and to study the composition of soil and endophytic bacterial communities as an effect of addition of structurally related phenolic compounds (SA and MCPA). However, to disentangle the mechanisms behind the shifts in bacterial communities and their functions, additional research is needed.

## Data Availability Statement

The datasets presented in this study can be found in online repositories. The latest version of link to repository of our sequencing data is https://www.ncbi.nlm.nih.gov/bioproject/PRJNA809520.

## Author Contributions

EM, ST, MU, and JV designed the experimental setup and analysis and wrote the manuscript. EM and MU performed the experiments and prepared the samples. EM and ST prepared the samples for DNA extraction and sequencing and analyzed the results using bioinformatics tools. EM determined the fresh biomass of plants and analyzed the fresh biomass and MCPA removal data. KZ prepared the samples and measured the MCPA concentration in the soil. All authors agreed on the final version of the manuscript.

## Conflict of Interest

The authors declare that the research was conducted in the absence of any commercial or financial relationships that could be construed as a potential conflict of interest.

## Publisher’s Note

All claims expressed in this article are solely those of the authors and do not necessarily represent those of their affiliated organizations, or those of the publisher, the editors and the reviewers. Any product that may be evaluated in this article, or claim that may be made by its manufacturer, is not guaranteed or endorsed by the publisher.
