# Supplementary material for: The Effect of Syringic Acid and Phenoxy Herbicide 4-chloro-2-methylphenoxyacetic acid (MCPA) on Soil, Rhizosphere, and Plant Endosphere Microbiome
Source: Front Plant Sci. 2022 May 31;13:882228. doi: 10.3389/fpls.2022.882228 (PMC9195007; doi:10.3389/fpls.2022.882228)
Supplement: Supplementary file 1 [file Table_1.DOCX]

Supplementary Material

# Supplementary Tables

**Table S1**. Chromatographic conditions

| **Chromatographic conditions for MCPA, MCPA- D6** | | | | |
| --- | --- | --- | --- | --- |
| HPLC system | Series 1260 HPLC (Agilent Technologies)  Degasser (G1322A)  Binary Pump (G1312B)  High Performance Autosampler (G1367E)  Thermostatted Column Compartment (G1316A) | | | |
| Pre-Column | Agilent 1290 Infinity In-Line Filter (PN: 5067-4368) with 0.3μm frit ring installed (PN: 5023-0271) | | | |
| Column | Acquity UPLC BEH C18,1.7 μm; 2.1 x 100 mm | | | |
| Column oven temperature | 45°C | | | |
| Injection volume | 5 μL | | | |
| Mobile phase | Eluent A: 0.01% (v/v) acetic acid in water (with 5% acetonitrile) Eluent B: 0.01% (v/v) acetic acid in acetonitrile | | | |
| Gradient | Time [min] | % Eluent A | % Eluent B | Flow [mL/min] |
|  | 0 | 80 | 20 | 0.3 |
|  | 4 | 70 | 30 | 0.3 |
|  | 7 | 10 | 90 | 0.3 |
|  | 8.5 | 10 | 90 | 0.3 |
|  | 8.6 | 80 | 20 | 0.3 |
|  | 13 | 80 | 20 | 0.3 |
| Total analysis time | Stop time 13 min | | Post time 1 min | |
| Retention time (approx.) | 5.9 min for MCPA | | 5.8 min for MCPA- D6 | |

**Table S2.** Mass spectrometric conditions

| **Mass spectrometric conditions for MCPA, MCPA- D6** | | | | | | |
| --- | --- | --- | --- | --- | --- | --- |
| MS system | Agilent Technologies 6460A Triple Quad LC/MS | | | | | |
| Ionisation type | Electrospray (ESI, Agilent Jet Stream, G1958-65138) | | | | | |
| Polarity | Negative ion mode | | | | | |
| Drying Gas Temperature | 250 °C | | | | | |
| Drying gas flow | 8 (l/min) | | | | | |
| Sheath Gas Heater | 350 °C | | | | | |
| Sheath Gas Flow | 11 (l/min) | | | | | |
| Nebulizer pressure | 40 (psi) | | | | | |
| Capillary voltage | 2000 (V) | | | | | |
| Nozzle voltage | 500 (V) | | | | | |
| Scan type | MS/MS, Multiple Reaction Monitoring (MRM) | | | | | |
| Time segments | Index | Start Time (min) | | Divert Valve | | Delta EMV |
|  | 1 | 0 | | To Waste | | 0 |
|  | 2 | 4 | | To MS | | 300 |
|  | 3 | 8 | | To Waste | | 0 |
| Scan resolution (FWHM) | MS1- Unit (0.7 amu) | | | MS2- Unit (0.7 amu) | | |
| Analyte monitored | Ion mass transition monitored (m/z) | | Fragmentor (V) | | Collision  energy (V) | Cell AcceleratorVoltage |
| MCPA | 199.1→141.0 | | 80 | | 12 | 4 |
|  | 201.1→143.0 | | 80 | | 8 | 4 |
| MCPA -D6 | 205.1→147.1 | | 56 | | 8 | 4 |
|  | 207.1→149.1 | | 56 | | 8 | 4 |

Table S3. Submitted samples to the NCBI BioSample database (Sequence Read Archive, SRA) (project accession number: PRJNA809520)

| **sample_name** | **organism** | **collection_date** | **env_broad_scale*** | **env_local_scale*** | **env_medium*** | **geo_loc_name*** | **host** | **lat_lon*** | **herbicide_regm*** | **host_phenotype** | **humidity_regm*** | **plant_body_site** | **description** |
| --- | --- | --- | --- | --- | --- | --- | --- | --- | --- | --- | --- | --- | --- |
| Bs_t1 | Cucurbita pepo cv Atena Polka | 2021-05 | soil | not applicable | Poland: Lodz, greenhouse experiment | not applicable | Cucurbita pepo | not applicable | not applicable | Bs_t1 | 60% v/w, watered daily | bulk soil | Bulksoil_control_t1 |
| Bs_t2 | Cucurbita pepo cv Atena Polka | 2021-05 | soil | not applicable | Poland: Lodz, greenhouse experiment | not applicable | Cucurbita pepo | not applicable | not applicable | Bs_t2 | 60% v/w, watered daily | bulk soil | Bulksoil_control_t2 |
| Bs_t3 | Cucurbita pepo cv Atena Polka | 2021-05 | soil | not applicable | Poland: Lodz, greenhouse experiment | not applicable | Cucurbita pepo | not applicable | not applicable | Bs_t3 | 60% v/w, watered daily | bulk soil | Bulksoil_control_t3 |
| Bs_s1 | Cucurbita pepo cv Atena Polka | 2021-05 | soil | not applicable | Poland: Lodz, greenhouse experiment | not applicable | Cucurbita pepo | not applicable | not applicable | Bs_s1 | 60% v/w, watered daily | bulk soil | Bulksoil_control_s1 |
| Bs_s2 | Cucurbita pepo cv Atena Polka | 2021-05 | soil | not applicable | Poland: Lodz, greenhouse experiment | not applicable | Cucurbita pepo | not applicable | not applicable | Bs_s2 | 60% v/w, watered daily | bulk soil | Bulksoil_control_s2 |
| Bs_s3 | Cucurbita pepo cv Atena Polka | 2021-05 | soil | not applicable | Poland: Lodz, greenhouse experiment | not applicable | Cucurbita pepo | not applicable | not applicable | Bs_s3 | 60% v/w, watered daily | bulk soil | Bulksoil_control_s3 |
| Bs_sa_t1 | Cucurbita pepo cv Atena Polka | 2021-05 | soil | not applicable | Poland: Lodz, greenhouse experiment | not applicable | Cucurbita pepo | not applicable | not applicable | Bs_sa_t1 | 60% v/w, watered daily | bulk soil | Bulksoil_syryngicacid_t1 |
| Bs_sa_t2 | Cucurbita pepo cv Atena Polka | 2021-05 | soil | not applicable | Poland: Lodz, greenhouse experiment | not applicable | Cucurbita pepo | not applicable | not applicable | Bs_sa_t2 | 60% v/w, watered daily | bulk soil | Bulksoil_syryngicacid_t2 |
| Bs_sa_t3 | Cucurbita pepo cv Atena Polka | 2021-05 | soil | not applicable | Poland: Lodz, greenhouse experiment | not applicable | Cucurbita pepo | not applicable | not applicable | Bs_sa_t3 | 60% v/w, watered daily | bulk soil | Bulksoil_syryngicacid_t3 |
| Bs_sa_s1 | Cucurbita pepo cv Atena Polka | 2021-05 | soil | not applicable | Poland: Lodz, greenhouse experiment | not applicable | Cucurbita pepo | not applicable | not applicable | Bs_sa_s1 | 60% v/w, watered daily | bulk soil | Bulksoil_syryngicacid_s1 |
| Bs_sa_s2 | Cucurbita pepo cv Atena Polka | 2021-05 | soil | not applicable | Poland: Lodz, greenhouse experiment | not applicable | Cucurbita pepo | not applicable | not applicable | Bs_sa_s2 | 60% v/w, watered daily | bulk soil | Bulksoil_syryngicacid_s2 |
| Bs_sa_s3 | Cucurbita pepo cv Atena Polka | 2021-05 | soil | not applicable | Poland: Lodz, greenhouse experiment | not applicable | Cucurbita pepo | not applicable | not applicable | Bs_sa_s3 | 60% v/w, watered daily | bulk soil | Bulksoil_syryngicacid_s3 |
| Bs_mcpa_t1 | Cucurbita pepo cv Atena Polka | 2021-05 | soil | not applicable | Poland: Lodz, greenhouse experiment | not applicable | Cucurbita pepo | not applicable | herbicide: MCPA (0.05mM) applied at the beggining of experiment before planting the seedling | Bs_mcpa_t1 | 60% v/w, watered daily | bulk soil | Bulksoil_mcpa_t1 |
| Bs_mcpa_t2 | Cucurbita pepo cv Atena Polka | 2021-05 | soil | not applicable | Poland: Lodz, greenhouse experiment | not applicable | Cucurbita pepo | not applicable | herbicide: MCPA (0.05mM) applied at the beggining of experiment before planting the seedling | Bs_mcpa_t2 | 60% v/w, watered daily | bulk soil | Bulksoil_mcpa_t2 |
| Bs_mcpa_t3 | Cucurbita pepo cv Atena Polka | 2021-05 | soil | not applicable | Poland: Lodz, greenhouse experiment | not applicable | Cucurbita pepo | not applicable | herbicide: MCPA (0.05mM) applied at the beggining of experiment before planting the seedling | Bs_mcpa_t3 | 60% v/w, watered daily | bulk soil | Bulksoil_mcpa_t3 |
| Bs_mcpa_s1 | Cucurbita pepo cv Atena Polka | 2021-05 | soil | not applicable | Poland: Lodz, greenhouse experiment | not applicable | Cucurbita pepo | not applicable | herbicide: MCPA (0.05mM) applied at the beggining of experiment before planting the seedling | Bs_mcpa_s1 | 60% v/w, watered daily | bulk soil | Bulksoil_mcpa_s1 |
| Bs_mcpa_s2 | Cucurbita pepo cv Atena Polka | 2021-05 | soil | not applicable | Poland: Lodz, greenhouse experiment | not applicable | Cucurbita pepo | not applicable | herbicide: MCPA (0.05mM) applied at the beggining of experiment before planting the seedling | Bs_mcpa_s2 | 60% v/w, watered daily | bulk soil | Bulksoil_mcpa_s2 |
| Bs_mcpa_s3 | Cucurbita pepo cv Atena Polka | 2021-05 | soil | not applicable | Poland: Lodz, greenhouse experiment | not applicable | Cucurbita pepo | not applicable | herbicide: MCPA (0.05mM) applied at the beggining of experiment before planting the seedling | Bs_mcpa_s3 | 60% v/w, watered daily | bulk soil | Bulksoil_mcpa_s3 |
| Bs_sa_mcpa_t1 | Cucurbita pepo cv Atena Polka | 2021-05 | soil | not applicable | Poland: Lodz, greenhouse experiment | not applicable | Cucurbita pepo | not applicable | herbicide: MCPA (0.05mM) applied at the beggining of experiment before planting the seedling | Bs_sa_mcpa_t1 | 60% v/w, watered daily | bulk soil | Bulksoil_syryngicacid_mcpa_t1 |
| Bs_sa_mcpa_t2 | Cucurbita pepo cv Atena Polka | 2021-05 | soil | not applicable | Poland: Lodz, greenhouse experiment | not applicable | Cucurbita pepo | not applicable | herbicide: MCPA (0.05mM) applied at the beggining of experiment before planting the seedling | Bs_sa_mcpa_t2 | 60% v/w, watered daily | bulk soil | Bulksoil_syryngicacid_mcpa_t2 |
| Bs_sa_mcpa_t3 | Cucurbita pepo cv Atena Polka | 2021-05 | soil | not applicable | Poland: Lodz, greenhouse experiment | not applicable | Cucurbita pepo | not applicable | herbicide: MCPA (0.05mM) applied at the beggining of experiment before planting the seedling | Bs_sa_mcpa_t3 | 60% v/w, watered daily | bulk soil | Bulksoil_syryngicacid_mcpa_t3 |
| Bs_sa_mcpa_s1 | Cucurbita pepo cv Atena Polka | 2021-05 | soil | not applicable | Poland: Lodz, greenhouse experiment | not applicable | Cucurbita pepo | not applicable | herbicide: MCPA (0.05mM) applied at the beggining of experiment before planting the seedling | Bs_sa_mcpa_s1 | 60% v/w, watered daily | bulk soil | Bulksoil_syryngicacid_mcpa_s1 |
| Bs_sa_mcpa_s2 | Cucurbita pepo cv Atena Polka | 2021-05 | soil | not applicable | Poland: Lodz, greenhouse experiment | not applicable | Cucurbita pepo | not applicable | herbicide: MCPA (0.05mM) applied at the beggining of experiment before planting the seedling | Bs_sa_mcpa_s2 | 60% v/w, watered daily | bulk soil | Bulksoil_syryngicacid_mcpa_s2 |
| Bs_sa_mcpa_s3 | Cucurbita pepo cv Atena Polka | 2021-05 | soil | not applicable | Poland: Lodz, greenhouse experiment | not applicable | Cucurbita pepo | not applicable | herbicide: MCPA (0.05mM) applied at the beggining of experiment before planting the seedling | Bs_sa_mcpa_s3 | 60% v/w, watered daily | bulk soil | Bulksoil_syryngicacid_mcpa_s3 |
| Rh_t1 | Cucurbita pepo cv Atena Polka | 2021-05 | rhizospheric soil | not applicable | Poland: Lodz, greenhouse experiment | not applicable | Cucurbita pepo | not applicable | not applicable | Rh_t1 | 60% v/w, watered daily | rhizospheric soil | Rhizosphericsoil_control_t1 |
| Rh_t2 | Cucurbita pepo cv Atena Polka | 2021-05 | rhizospheric soil | not applicable | Poland: Lodz, greenhouse experiment | not applicable | Cucurbita pepo | not applicable | not applicable | Rh_t2 | 60% v/w, watered daily | rhizospheric soil | Rhizosphericsoil_control_t2 |
| Rh_t3 | Cucurbita pepo cv Atena Polka | 2021-05 | rhizospheric soil | not applicable | Poland: Lodz, greenhouse experiment | not applicable | Cucurbita pepo | not applicable | not applicable | Rh_t3 | 60% v/w, watered daily | rhizospheric soil | Rhizosphericsoil_control_t3 |
| Rh_s1 | Cucurbita pepo cv Atena Polka | 2021-05 | rhizospheric soil | not applicable | Poland: Lodz, greenhouse experiment | not applicable | Cucurbita pepo | not applicable | not applicable | Rh_s1 | 60% v/w, watered daily | rhizospheric soil | Rhizosphericsoil_control_s1 |
| Rh_s2 | Cucurbita pepo cv Atena Polka | 2021-05 | rhizospheric soil | not applicable | Poland: Lodz, greenhouse experiment | not applicable | Cucurbita pepo | not applicable | not applicable | Rh_s2 | 60% v/w, watered daily | rhizospheric soil | Rhizosphericsoil_control_s2 |
| Rh_s3 | Cucurbita pepo cv Atena Polka | 2021-05 | rhizospheric soil | not applicable | Poland: Lodz, greenhouse experiment | not applicable | Cucurbita pepo | not applicable | not applicable | Rh_s3 | 60% v/w, watered daily | rhizospheric soil | Rhizosphericsoil_control_s3 |
| Rh_sa_t1 | Cucurbita pepo cv Atena Polka | 2021-05 | rhizospheric soil | not applicable | Poland: Lodz, greenhouse experiment | not applicable | Cucurbita pepo | not applicable | not applicable | Rh_sa_t1 | 60% v/w, watered daily | rhizospheric soil | Rhizosphericsoil_syryngicacid_t1 |
| Rh_sa_t2 | Cucurbita pepo cv Atena Polka | 2021-05 | rhizospheric soil | not applicable | Poland: Lodz, greenhouse experiment | not applicable | Cucurbita pepo | not applicable | not applicable | Rh_sa_t2 | 60% v/w, watered daily | rhizospheric soil | Rhizosphericsoil_syryngicacid_t2 |
| Rh_sa_t3 | Cucurbita pepo cv Atena Polka | 2021-05 | rhizospheric soil | not applicable | Poland: Lodz, greenhouse experiment | not applicable | Cucurbita pepo | not applicable | not applicable | Rh_sa_t3 | 60% v/w, watered daily | rhizospheric soil | Rhizosphericsoil_syryngicacid_t3 |
| Rh_sa_s1 | Cucurbita pepo cv Atena Polka | 2021-05 | rhizospheric soil | not applicable | Poland: Lodz, greenhouse experiment | not applicable | Cucurbita pepo | not applicable | not applicable | Rh_sa_s1 | 60% v/w, watered daily | rhizospheric soil | Rhizosphericsoil_syryngicacid_s1 |
| Rh_sa_s2 | Cucurbita pepo cv Atena Polka | 2021-05 | rhizospheric soil | not applicable | Poland: Lodz, greenhouse experiment | not applicable | Cucurbita pepo | not applicable | not applicable | Rh_sa_s2 | 60% v/w, watered daily | rhizospheric soil | Rhizosphericsoil_syryngicacid_s2 |
| Rh_sa_s3 | Cucurbita pepo cv Atena Polka | 2021-05 | rhizospheric soil | not applicable | Poland: Lodz, greenhouse experiment | not applicable | Cucurbita pepo | not applicable | not applicable | Rh_sa_s3 | 60% v/w, watered daily | rhizospheric soil | Rhizosphericsoil_syryngicacid_s3 |
| Rh_mcpa_t1 | Cucurbita pepo cv Atena Polka | 2021-05 | rhizospheric soil | not applicable | Poland: Lodz, greenhouse experiment | not applicable | Cucurbita pepo | not applicable | herbicide: MCPA (0.05mM) applied at the beggining of experiment before planting the seedling | Rh_mcpa_t1 | 60% v/w, watered daily | rhizospheric soil | Rhizosphericsoil_mcpa_t1 |
| Rh_mcpa_t2 | Cucurbita pepo cv Atena Polka | 2021-05 | rhizospheric soil | not applicable | Poland: Lodz, greenhouse experiment | not applicable | Cucurbita pepo | not applicable | herbicide: MCPA (0.05mM) applied at the beggining of experiment before planting the seedling | Rh_mcpa_t2 | 60% v/w, watered daily | rhizospheric soil | Rhizosphericsoil_mcpa_t2 |
| Rh_mcpa_t3 | Cucurbita pepo cv Atena Polka | 2021-05 | rhizospheric soil | not applicable | Poland: Lodz, greenhouse experiment | not applicable | Cucurbita pepo | not applicable | herbicide: MCPA (0.05mM) applied at the beggining of experiment before planting the seedling | Rh_mcpa_t3 | 60% v/w, watered daily | rhizospheric soil | Rhizosphericsoil_mcpa_t3 |
| Rh_mcpa_s1 | Cucurbita pepo cv Atena Polka | 2021-05 | rhizospheric soil | not applicable | Poland: Lodz, greenhouse experiment | not applicable | Cucurbita pepo | not applicable | herbicide: MCPA (0.05mM) applied at the beggining of experiment before planting the seedling | Rh_mcpa_s1 | 60% v/w, watered daily | rhizospheric soil | Rhizosphericsoil_mcpa_s1 |
| Rh_mcpa_s2 | Cucurbita pepo cv Atena Polka | 2021-05 | rhizospheric soil | not applicable | Poland: Lodz, greenhouse experiment | not applicable | Cucurbita pepo | not applicable | herbicide: MCPA (0.05mM) applied at the beggining of experiment before planting the seedling | Rh_mcpa_s2 | 60% v/w, watered daily | rhizospheric soil | Rhizosphericsoil_mcpa_s2 |
| Rh_mcpa_s3 | Cucurbita pepo cv Atena Polka | 2021-05 | rhizospheric soil | not applicable | Poland: Lodz, greenhouse experiment | not applicable | Cucurbita pepo | not applicable | herbicide: MCPA (0.05mM) applied at the beggining of experiment before planting the seedling | Rh_mcpa_s3 | 60% v/w, watered daily | rhizospheric soil | Rhizosphericsoil_mcpa_s3 |
| Rh_mcpa_sa_t1 | Cucurbita pepo cv Atena Polka | 2021-05 | rhizospheric soil | not applicable | Poland: Lodz, greenhouse experiment | not applicable | Cucurbita pepo | not applicable | herbicide: MCPA (0.05mM) applied at the beggining of experiment before planting the seedling | Rh_mcpa_sa_t1 | 60% v/w, watered daily | rhizospheric soil | Rhizosphericsoil_mcpa_syryngicacid_t1 |
| Rh_mcpa_sa_t2 | Cucurbita pepo cv Atena Polka | 2021-05 | rhizospheric soil | not applicable | Poland: Lodz, greenhouse experiment | not applicable | Cucurbita pepo | not applicable | herbicide: MCPA (0.05mM) applied at the beggining of experiment before planting the seedling | Rh_mcpa_sa_t2 | 60% v/w, watered daily | rhizospheric soil | Rhizosphericsoil_mcpa_syryngicacid_t2 |
| Rh_mcpa_sa_t3 | Cucurbita pepo cv Atena Polka | 2021-05 | rhizospheric soil | not applicable | Poland: Lodz, greenhouse experiment | not applicable | Cucurbita pepo | not applicable | herbicide: MCPA (0.05mM) applied at the beggining of experiment before planting the seedling | Rh_mcpa_sa_t3 | 60% v/w, watered daily | rhizospheric soil | Rhizosphericsoil_mcpa_syryngicacid_t3 |
| Rh_mcpa_sa_s1 | Cucurbita pepo cv Atena Polka | 2021-05 | rhizospheric soil | not applicable | Poland: Lodz, greenhouse experiment | not applicable | Cucurbita pepo | not applicable | herbicide: MCPA (0.05mM) applied at the beggining of experiment before planting the seedling | Rh_mcpa_sa_s1 | 60% v/w, watered daily | rhizospheric soil | Rhizosphericsoil_mcpa_syryngicacid_s1 |
| Rh_mcpa_sa_s2 | Cucurbita pepo cv Atena Polka | 2021-05 | rhizospheric soil | not applicable | Poland: Lodz, greenhouse experiment | not applicable | Cucurbita pepo | not applicable | herbicide: MCPA (0.05mM) applied at the beggining of experiment before planting the seedling | Rh_mcpa_sa_s2 | 60% v/w, watered daily | rhizospheric soil | Rhizosphericsoil_mcpa_syryngicacid_s2 |
| Rh_mcpa_sa_s3 | Cucurbita pepo cv Atena Polka | 2021-05 | rhizospheric soil | not applicable | Poland: Lodz, greenhouse experiment | not applicable | Cucurbita pepo | not applicable | herbicide: MCPA (0.05mM) applied at the beggining of experiment before planting the seedling | Rh_mcpa_sa_s3 | 60% v/w, watered daily | rhizospheric soil | Rhizosphericsoil_mcpa_syryngicacid_s3 |
| Ro_t1 | Cucurbita pepo cv Atena Polka | 2021-05 | root | not applicable | Poland: Lodz, greenhouse experiment | not applicable | Cucurbita pepo | not applicable | not applicable | Ro_t1 | 60% v/w, watered daily | root | root_control_t1 |
| Ro_t2 | Cucurbita pepo cv Atena Polka | 2021-05 | root | not applicable | Poland: Lodz, greenhouse experiment | not applicable | Cucurbita pepo | not applicable | not applicable | Ro_t2 | 60% v/w, watered daily | root | root_control_t2 |
| Ro_t3 | Cucurbita pepo cv Atena Polka | 2021-05 | root | not applicable | Poland: Lodz, greenhouse experiment | not applicable | Cucurbita pepo | not applicable | not applicable | Ro_t3 | 60% v/w, watered daily | root | root_control_t3 |
| Ro_s1 | Cucurbita pepo cv Atena Polka | 2021-05 | root | not applicable | Poland: Lodz, greenhouse experiment | not applicable | Cucurbita pepo | not applicable | not applicable | Ro_s1 | 60% v/w, watered daily | root | root_control_s1 |
| Ro_s2 | Cucurbita pepo cv Atena Polka | 2021-05 | root | not applicable | Poland: Lodz, greenhouse experiment | not applicable | Cucurbita pepo | not applicable | not applicable | Ro_s2 | 60% v/w, watered daily | root | root_control_s2 |
| Ro_s3 | Cucurbita pepo cv Atena Polka | 2021-05 | root | not applicable | Poland: Lodz, greenhouse experiment | not applicable | Cucurbita pepo | not applicable | not applicable | Ro_s3 | 60% v/w, watered daily | root | root_control_s3 |
| Ro_sa_t1 | Cucurbita pepo cv Atena Polka | 2021-05 | root | not applicable | Poland: Lodz, greenhouse experiment | not applicable | Cucurbita pepo | not applicable | not applicable | Ro_sa_t1 | 60% v/w, watered daily | root | root_syryngicacid_t1 |
| Ro_sa_t2 | Cucurbita pepo cv Atena Polka | 2021-05 | root | not applicable | Poland: Lodz, greenhouse experiment | not applicable | Cucurbita pepo | not applicable | not applicable | Ro_sa_t2 | 60% v/w, watered daily | root | root_syryngicacid_t2 |
| Ro_sa_t3 | Cucurbita pepo cv Atena Polka | 2021-05 | root | not applicable | Poland: Lodz, greenhouse experiment | not applicable | Cucurbita pepo | not applicable | not applicable | Ro_sa_t3 | 60% v/w, watered daily | root | root_syryngicacid_t3 |
| Ro_sa_s1 | Cucurbita pepo cv Atena Polka | 2021-05 | root | not applicable | Poland: Lodz, greenhouse experiment | not applicable | Cucurbita pepo | not applicable | not applicable | Ro_sa_s1 | 60% v/w, watered daily | root | root_syryngicacid_s1 |
| Ro_sa_s2 | Cucurbita pepo cv Atena Polka | 2021-05 | root | not applicable | Poland: Lodz, greenhouse experiment | not applicable | Cucurbita pepo | not applicable | not applicable | Ro_sa_s2 | 60% v/w, watered daily | root | root_syryngicacid_s2 |
| Ro_sa_s3 | Cucurbita pepo cv Atena Polka | 2021-05 | root | not applicable | Poland: Lodz, greenhouse experiment | not applicable | Cucurbita pepo | not applicable | not applicable | Ro_sa_s3 | 60% v/w, watered daily | root | root_syryngicacid_s3 |
| Ro_mcpa_t1 | Cucurbita pepo cv Atena Polka | 2021-05 | root | not applicable | Poland: Lodz, greenhouse experiment | not applicable | Cucurbita pepo | not applicable | herbicide: MCPA (0.05mM) applied at the beggining of experiment before planting the seedling | Ro_mcpa_t1 | 60% v/w, watered daily | root | root_mcpa_t1 |
| Ro_mcpa_t2 | Cucurbita pepo cv Atena Polka | 2021-05 | root | not applicable | Poland: Lodz, greenhouse experiment | not applicable | Cucurbita pepo | not applicable | herbicide: MCPA (0.05mM) applied at the beggining of experiment before planting the seedling | Ro_mcpa_t2 | 60% v/w, watered daily | root | root_mcpa_t2 |
| Ro_mcpa_t3 | Cucurbita pepo cv Atena Polka | 2021-05 | root | not applicable | Poland: Lodz, greenhouse experiment | not applicable | Cucurbita pepo | not applicable | herbicide: MCPA (0.05mM) applied at the beggining of experiment before planting the seedling | Ro_mcpa_t3 | 60% v/w, watered daily | root | rootot_mcpa_t3 |
| Ro_mcpa_s1 | Cucurbita pepo cv Atena Polka | 2021-05 | root | not applicable | Poland: Lodz, greenhouse experiment | not applicable | Cucurbita pepo | not applicable | herbicide: MCPA (0.05mM) applied at the beggining of experiment before planting the seedling | Ro_mcpa_s1 | 60% v/w, watered daily | root | root_mcpa_s1 |
| Ro_mcpa_s2 | Cucurbita pepo cv Atena Polka | 2021-05 | root | not applicable | Poland: Lodz, greenhouse experiment | not applicable | Cucurbita pepo | not applicable | herbicide: MCPA (0.05mM) applied at the beggining of experiment before planting the seedling | Ro_mcpa_s2 | 60% v/w, watered daily | root | root_mcpa_s2 |
| Ro_mcpa_s3 | Cucurbita pepo cv Atena Polka | 2021-05 | root | not applicable | Poland: Lodz, greenhouse experiment | not applicable | Cucurbita pepo | not applicable | herbicide: MCPA (0.05mM) applied at the beggining of experiment before planting the seedling | Ro_mcpa_s3 | 60% v/w, watered daily | root | root_mcpa_s3 |
| Ro_sa_mcpa_t1 | Cucurbita pepo cv Atena Polka | 2021-05 | root | not applicable | Poland: Lodz, greenhouse experiment | not applicable | Cucurbita pepo | not applicable | herbicide: MCPA (0.05mM) applied at the beggining of experiment before planting the seedling | Ro_sa_mcpa_t1 | 60% v/w, watered daily | root | root_syryngicacid_mcpa_t1 |
| Ro_sa_mcpa_t2 | Cucurbita pepo cv Atena Polka | 2021-05 | root | not applicable | Poland: Lodz, greenhouse experiment | not applicable | Cucurbita pepo | not applicable | herbicide: MCPA (0.05mM) applied at the beggining of experiment before planting the seedling | Ro_sa_mcpa_t2 | 60% v/w, watered daily | root | root_syryngicacid_mcpa_t2 |
| Ro_sa_mcpa_t3 | Cucurbita pepo cv Atena Polka | 2021-05 | root | not applicable | Poland: Lodz, greenhouse experiment | not applicable | Cucurbita pepo | not applicable | herbicide: MCPA (0.05mM) applied at the beggining of experiment before planting the seedling | Ro_sa_mcpa_t3 | 60% v/w, watered daily | root | root_syryngicacid_mcpa_t3 |
| Ro_sa_mcpa_s1 | Cucurbita pepo cv Atena Polka | 2021-05 | root | not applicable | Poland: Lodz, greenhouse experiment | not applicable | Cucurbita pepo | not applicable | herbicide: MCPA (0.05mM) applied at the beggining of experiment before planting the seedling | Ro_sa_mcpa_s1 | 60% v/w, watered daily | root | root_syryngicacid_mcpa_s1 |
| Ro_sa_mcpa_s2 | Cucurbita pepo cv Atena Polka | 2021-05 | root | not applicable | Poland: Lodz, greenhouse experiment | not applicable | Cucurbita pepo | not applicable | herbicide: MCPA (0.05mM) applied at the beggining of experiment before planting the seedling | Ro_sa_mcpa_s2 | 60% v/w, watered daily | root | root_syryngicacid_mcpa_s2 |
| Ro_sa_mcpa_s3 | Cucurbita pepo cv Atena Polka | 2021-05 | root | not applicable | Poland: Lodz, greenhouse experiment | not applicable | Cucurbita pepo | not applicable | herbicide: MCPA (0.05mM) applied at the beggining of experiment before planting the seedling | Ro_sa_mcpa_s3 | 60% v/w, watered daily | root | root_syryngicacid_mcpa_s3 |
| Le_t1 | Cucurbita pepo cv Atena Polka | 2021-05 | leaf | not applicable | Poland: Lodz, greenhouse experiment | not applicable | Cucurbita pepo | not applicable | not applicable | Le_t1 | 60% v/w, watered daily | leaf | leaf_control_t1 |
| Le_t2 | Cucurbita pepo cv Atena Polka | 2021-05 | leaf | not applicable | Poland: Lodz, greenhouse experiment | not applicable | Cucurbita pepo | not applicable | not applicable | Le_t2 | 60% v/w, watered daily | leaf | leaf_control_t2 |
| Le_t3 | Cucurbita pepo cv Atena Polka | 2021-05 | leaf | not applicable | Poland: Lodz, greenhouse experiment | not applicable | Cucurbita pepo | not applicable | not applicable | Le_t3 | 60% v/w, watered daily | leaf | leaf_control_t3 |
| Le_s1 | Cucurbita pepo cv Atena Polka | 2021-05 | leaf | not applicable | Poland: Lodz, greenhouse experiment | not applicable | Cucurbita pepo | not applicable | not applicable | Le_s1 | 60% v/w, watered daily | leaf | leaf_control_s1 |
| Le_s2 | Cucurbita pepo cv Atena Polka | 2021-05 | leaf | not applicable | Poland: Lodz, greenhouse experiment | not applicable | Cucurbita pepo | not applicable | not applicable | Le_s2 | 60% v/w, watered daily | leaf | leaf_control_s2 |
| Le_s3 | Cucurbita pepo cv Atena Polka | 2021-05 | leaf | not applicable | Poland: Lodz, greenhouse experiment | not applicable | Cucurbita pepo | not applicable | not applicable | Le_s3 | 60% v/w, watered daily | leaf | leaf_control_s3 |
| Le_sa_t1 | Cucurbita pepo cv Atena Polka | 2021-05 | leaf | not applicable | Poland: Lodz, greenhouse experiment | not applicable | Cucurbita pepo | not applicable | not applicable | Le_sa_t1 | 60% v/w, watered daily | leaf | leaf_syryngicacid_t1 |
| Le_sa_t2 | Cucurbita pepo cv Atena Polka | 2021-05 | leaf | not applicable | Poland: Lodz, greenhouse experiment | not applicable | Cucurbita pepo | not applicable | not applicable | Le_sa_t2 | 60% v/w, watered daily | leaf | leaf_syryngicacid_t2 |
| Le_sa_t3 | Cucurbita pepo cv Atena Polka | 2021-05 | leaf | not applicable | Poland: Lodz, greenhouse experiment | not applicable | Cucurbita pepo | not applicable | not applicable | Le_sa_t3 | 60% v/w, watered daily | leaf | leaf_syryngicacid_t3 |
| Le_sa_s1 | Cucurbita pepo cv Atena Polka | 2021-05 | leaf | not applicable | Poland: Lodz, greenhouse experiment | not applicable | Cucurbita pepo | not applicable | not applicable | Le_sa_s1 | 60% v/w, watered daily | leaf | leaf_syryngicacid_s1 |
| Le_sa_s2 | Cucurbita pepo cv Atena Polka | 2021-05 | leaf | not applicable | Poland: Lodz, greenhouse experiment | not applicable | Cucurbita pepo | not applicable | not applicable | Le_sa_s2 | 60% v/w, watered daily | leaf | leaf_syryngicacid_s2 |
| Le_sa_s3 | Cucurbita pepo cv Atena Polka | 2021-05 | leaf | not applicable | Poland: Lodz, greenhouse experiment | not applicable | Cucurbita pepo | not applicable | not applicable | Le_sa_s3 | 60% v/w, watered daily | leaf | leaf_syryngicacid_s3 |
| Le_mcpa_t1 | Cucurbita pepo cv Atena Polka | 2021-05 | leaf | not applicable | Poland: Lodz, greenhouse experiment | not applicable | Cucurbita pepo | not applicable | herbicide: MCPA (0.05mM) applied at the beggining of experiment before planting the seedling | Le_mcpa_t1 | 60% v/w, watered daily | leaf | leaf_mcpa_t1 |
| Le_mcpa_t2 | Cucurbita pepo cv Atena Polka | 2021-05 | leaf | not applicable | Poland: Lodz, greenhouse experiment | not applicable | Cucurbita pepo | not applicable | herbicide: MCPA (0.05mM) applied at the beggining of experiment before planting the seedling | Le_mcpa_t2 | 60% v/w, watered daily | leaf | leaf_mcpa_t2 |
| Le_mcpa_t3 | Cucurbita pepo cv Atena Polka | 2021-05 | leaf | not applicable | Poland: Lodz, greenhouse experiment | not applicable | Cucurbita pepo | not applicable | herbicide: MCPA (0.05mM) applied at the beggining of experiment before planting the seedling | Le_mcpa_t3 | 60% v/w, watered daily | leaf | leaf_mcpa_t3 |
| Le_mcpa_s1 | Cucurbita pepo cv Atena Polka | 2021-05 | leaf | not applicable | Poland: Lodz, greenhouse experiment | not applicable | Cucurbita pepo | not applicable | herbicide: MCPA (0.05mM) applied at the beggining of experiment before planting the seedling | Le_mcpa_s1 | 60% v/w, watered daily | leaf | leaf_mcpa_s1 |
| Le_mcpa_s2 | Cucurbita pepo cv Atena Polka | 2021-05 | leaf | not applicable | Poland: Lodz, greenhouse experiment | not applicable | Cucurbita pepo | not applicable | herbicide: MCPA (0.05mM) applied at the beggining of experiment before planting the seedling | Le_mcpa_s2 | 60% v/w, watered daily | leaf | leaf_mcpa_s2 |
| Le_mcpa_s3 | Cucurbita pepo cv Atena Polka | 2021-05 | leaf | not applicable | Poland: Lodz, greenhouse experiment | not applicable | Cucurbita pepo | not applicable | herbicide: MCPA (0.05mM) applied at the beggining of experiment before planting the seedling | Le_mcpa_s3 | 60% v/w, watered daily | leaf | leaf_mcpa_s3 |
| Le_mcpa_sa_t1 | Cucurbita pepo cv Atena Polka | 2021-05 | leaf | not applicable | Poland: Lodz, greenhouse experiment | not applicable | Cucurbita pepo | not applicable | herbicide: MCPA (0.05mM) applied at the beggining of experiment before planting the seedling | Le_mcpa_sa_t1 | 60% v/w, watered daily | leaf | leaf_mcpa_syryngicacid_t1 |
| Le_mcpa_sa_t2 | Cucurbita pepo cv Atena Polka | 2021-05 | leaf | not applicable | Poland: Lodz, greenhouse experiment | not applicable | Cucurbita pepo | not applicable | herbicide: MCPA (0.05mM) applied at the beggining of experiment before planting the seedling | Le_mcpa_sa_t2 | 60% v/w, watered daily | leaf | leaf_mcpa_syryngicacid_t2 |
| Le_mcpa_sa_t3 | Cucurbita pepo cv Atena Polka | 2021-05 | leaf | not applicable | Poland: Lodz, greenhouse experiment | not applicable | Cucurbita pepo | not applicable | herbicide: MCPA (0.05mM) applied at the beggining of experiment before planting the seedling | Le_mcpa_sa_t3 | 60% v/w, watered daily | leaf | leaf_mcpa_syryngicacid_t3 |
| Le_mcpa_sa_s1 | Cucurbita pepo cv Atena Polka | 2021-05 | leaf | not applicable | Poland: Lodz, greenhouse experiment | not applicable | Cucurbita pepo | not applicable | herbicide: MCPA (0.05mM) applied at the beggining of experiment before planting the seedling | Le_mcpa_sa_s1 | 60% v/w, watered daily | leaf | leaf_mcpa_syryngicacid_s1 |
| Le_mcpa_sa_s2 | Cucurbita pepo cv Atena Polka | 2021-05 | leaf | not applicable | Poland: Lodz, greenhouse experiment | not applicable | Cucurbita pepo | not applicable | herbicide: MCPA (0.05mM) applied at the beggining of experiment before planting the seedling | Le_mcpa_sa_s2 | 60% v/w, watered daily | leaf | leaf_mcpa_syryngicacid_s2 |
| Le_mcpa_sa_s3 | Cucurbita pepo cv Atena Polka | 2021-05 | leaf | not applicable | Poland: Lodz, greenhouse experiment | not applicable | Cucurbita pepo | not applicable | herbicide: MCPA (0.05mM) applied at the beggining of experiment before planting the seedling | Le_mcpa_sa_s3 | 60% v/w, watered daily | leaf | leaf_mcpa_syryngicacid_s3 |
| EMDNABlank | not applicable | not applicable | not applicable | not applicable | not applicable | not applicable | not applicable | not applicable | not applicable | blank | not applicable | not applicable | blank sample |
| MockB | not applicable | not applicable | not applicable | not applicable | not applicable | not applicable | not applicable | not applicable | not applicable | mock community | not applicable | not applicable | mock community sample |

***env_broad_scale- major environment type; env_local_scale- environmental entities having causal influences upon the entity at time of sampling ; env_medium- identified material displacement at the time of sampling; geo_loc_name- geographical origin of the sample ; lat_lon- the geographical coordinates ; herbicide_regm- information about herbicide treatment ; humidity_regm- humidity regime**

**Table S4. Sequence Read Archive (SRA) metadata table**

| **biosample_accession** | **library_ID** | **title** | [**library_strategy**](file:///C:\Users\Ela%20Mierzejewska\Desktop\SRA_metadata_acc.xlsx#'Library and Platform terms'!A2) | [**library_source**](file:///C:\Users\Ela%20Mierzejewska\Desktop\SRA_metadata_acc.xlsx#'Library and Platform terms'!A27) | [**library_selection**](file:///C:\Users\Ela%20Mierzejewska\Desktop\SRA_metadata_acc.xlsx#'Library and Platform terms'!A36) | **library_layout** | [**platform**](file:///C:\Users\Ela%20Mierzejewska\Desktop\SRA_metadata_acc.xlsx#'Library and Platform terms'!A66) | **instrument_model** | **design_description** | **filetype** | **filename** | **filename2** |
| --- | --- | --- | --- | --- | --- | --- | --- | --- | --- | --- | --- | --- |
| Bs_t1 | EM_01 | 16S rRNA in unplanted soil | AMPLICON | METAGENOMIC | PCR | paired | ILLUMINA | Illumina MiSeq | 515F-806R primers | fastq | EM01Cobs_S1_L001_R1_001.fastq.gz | EM01Cobs_S1_L001_R2_001.fastq.gz |
| Bs_t2 | EM_02 | 16S rRNA in unplanted soil | AMPLICON | METAGENOMIC | PCR | paired | ILLUMINA | Illumina MiSeq | 515F-806R primers | fastq | EM02Cobs_S2_L001_R1_001.fastq.gz | EM02Cobs_S2_L001_R2_001.fastq.gz |
| Bs_t3 | EM_03 | 16S rRNA in unplanted soil | AMPLICON | METAGENOMIC | PCR | paired | ILLUMINA | Illumina MiSeq | 515F-806R primers | fastq | EM03Cobs_S3_L001_R1_001.fastq.gz | EM03Cobs_S3_L001_R2_001.fastq.gz |
| Bs_s1 | EM_04 | 16S rRNA in unplanted soil | AMPLICON | METAGENOMIC | PCR | paired | ILLUMINA | Illumina MiSeq | 515F-806R primers | fastq | EM04Cobs_S4_L001_R1_001.fastq.gz | EM04Cobs_S4_L001_R2_001.fastq.gz |
| Bs_s2 | EM_05 | 16S rRNA in unplanted soil | AMPLICON | METAGENOMIC | PCR | paired | ILLUMINA | Illumina MiSeq | 515F-806R primers | fastq | EM05Cobs_S5_L001_R1_001.fastq.gz | EM05Cobs_S5_L001_R2_001.fastq.gz |
| Bs_s3 | EM_06 | 16S rRNA in unplanted soil | AMPLICON | METAGENOMIC | PCR | paired | ILLUMINA | Illumina MiSeq | 515F-806R primers | fastq | EM06Cobs_S6_L001_R1_001.fastq.gz | EM06Cobs_S6_L001_R2_001.fastq.gz |
| Bs_sa_t1 | EM_07 | 16S rRNA in unplanted soil treated with syringic acid | AMPLICON | METAGENOMIC | PCR | paired | ILLUMINA | Illumina MiSeq | 515F-806R primers | fastq | EM07CoSAbs_S7_L001_R1_001.fastq.gz | EM07CoSAbs_S7_L001_R2_001.fastq.gz |
| Bs_sa_t2 | EM_08 | 16S rRNA in unplanted soil treated with syringic acid | AMPLICON | METAGENOMIC | PCR | paired | ILLUMINA | Illumina MiSeq | 515F-806R primers | fastq | EM08CoSAbs_S8_L001_R1_001.fastq.gz | EM08CoSAbs_S8_L001_R2_001.fastq.gz |
| Bs_sa_t3 | EM_09 | 16S rRNA in unplanted soil treated with syringic acid | AMPLICON | METAGENOMIC | PCR | paired | ILLUMINA | Illumina MiSeq | 515F-806R primers | fastq | EM09CoSAbs_S9_L001_R1_001.fastq.gz | EM09CoSAbs_S9_L001_R2_001.fastq.gz |
| Bs_sa_s1 | EM_10 | 16S rRNA in unplanted soil treated with syringic acid | AMPLICON | METAGENOMIC | PCR | paired | ILLUMINA | Illumina MiSeq | 515F-806R primers | fastq | EM10CoSAbs_S10_L001_R1_001.fastq.gz | EM10CoSAbs_S10_L001_R2_001.fastq.gz |
| Bs_sa_s2 | EM_11 | 16S rRNA in unplanted soil treated with syringic acid | AMPLICON | METAGENOMIC | PCR | paired | ILLUMINA | Illumina MiSeq | 515F-806R primers | fastq | EM11CoSAbs_S11_L001_R1_001.fastq.gz | EM11CoSAbs_S11_L001_R2_001.fastq.gz |
| Bs_sa_s3 | EM_12 | 16S rRNA in unplanted soil treated with syringic acid | AMPLICON | METAGENOMIC | PCR | paired | ILLUMINA | Illumina MiSeq | 515F-806R primers | fastq | EM12CoSAbs_S12_L001_R1_001.fastq.gz | EM12CoSAbs_S12_L001_R2_001.fastq.gz |
| Bs_mcpa_t1 | EM_13 | 16S rRNA in unplanted soil treated with MCPA | AMPLICON | METAGENOMIC | PCR | paired | ILLUMINA | Illumina MiSeq | 515F-806R primers | fastq | EM13CoMCPAbs_S13_L001_R1_001.fastq.gz | EM13CoMCPAbs_S13_L001_R2_001.fastq.gz |
| Bs_mcpa_t2 | EM_14 | 16S rRNA in unplanted soil treated with MCPA | AMPLICON | METAGENOMIC | PCR | paired | ILLUMINA | Illumina MiSeq | 515F-806R primers | fastq | EM14CoMCPAbs_S14_L001_R1_001.fastq.gz | EM14CoMCPAbs_S14_L001_R2_001.fastq.gz |
| Bs_mcpa_t3 | EM_15 | 16S rRNA in unplanted soil treated with MCPA | AMPLICON | METAGENOMIC | PCR | paired | ILLUMINA | Illumina MiSeq | 515F-806R primers | fastq | EM15CoMCPAbs_S15_L001_R1_001.fastq.gz | EM15CoMCPAbs_S15_L001_R2_001.fastq.gz |
| Bs_mcpa_s1 | EM_16 | 16S rRNA in unplanted soil treated with MCPA | AMPLICON | METAGENOMIC | PCR | paired | ILLUMINA | Illumina MiSeq | 515F-806R primers | fastq | EM16CoMCPAbs_S16_L001_R1_001.fastq.gz | EM16CoMCPAbs_S16_L001_R2_001.fastq.gz |
| Bs_mcpa_s2 | EM_17 | 16S rRNA in unplanted soil treated with MCPA | AMPLICON | METAGENOMIC | PCR | paired | ILLUMINA | Illumina MiSeq | 515F-806R primers | fastq | EM17CoMCPAbs_S17_L001_R1_001.fastq.gz | EM17CoMCPAbs_S17_L001_R2_001.fastq.gz |
| Bs_mcpa_s3 | EM_18 | 16S rRNA in unplanted soil treated with MCPA | AMPLICON | METAGENOMIC | PCR | paired | ILLUMINA | Illumina MiSeq | 515F-806R primers | fastq | EM18CoMCPAbs_S18_L001_R1_001.fastq.gz | EM18CoMCPAbs_S18_L001_R2_001.fastq.gz |
| Bs_sa_mcpa_t1 | EM_19 | 16S rRNA in unplanted soil treated with syringic acid and MCPA | AMPLICON | METAGENOMIC | PCR | paired | ILLUMINA | Illumina MiSeq | 515F-806R primers | fastq | EM19CoMCPASAbs_S19_L001_R1_001.fastq.gz | EM19CoMCPASAbs_S19_L001_R2_001.fastq.gz |
| Bs_sa_mcpa_t2 | EM_20 | 16S rRNA in unplanted soil treated with syringic acid and MCPA | AMPLICON | METAGENOMIC | PCR | paired | ILLUMINA | Illumina MiSeq | 515F-806R primers | fastq | EM20CoMCPASAbs_S20_L001_R1_001.fastq.gz | EM20CoMCPASAbs_S20_L001_R2_001.fastq.gz |
| Bs_sa_mcpa_t3 | EM_21 | 16S rRNA in unplanted soil treated with syringic acid and MCPA | AMPLICON | METAGENOMIC | PCR | paired | ILLUMINA | Illumina MiSeq | 515F-806R primers | fastq | EM21CoMCPASAbs_S21_L001_R1_001.fastq.gz | EM21CoMCPASAbs_S21_L001_R2_001.fastq.gz |
| Bs_sa_mcpa_s1 | EM_22 | 16S rRNA in unplanted soil treated with syringic acid and MCPA | AMPLICON | METAGENOMIC | PCR | paired | ILLUMINA | Illumina MiSeq | 515F-806R primers | fastq | EM22CoMCPASAbs_S22_L001_R1_001.fastq.gz | EM22CoMCPASAbs_S22_L001_R2_001.fastq.gz |
| Bs_sa_mcpa_s2 | EM_23 | 16S rRNA in unplanted soil treated with syringic acid and MCPA | AMPLICON | METAGENOMIC | PCR | paired | ILLUMINA | Illumina MiSeq | 515F-806R primers | fastq | EM23CoMCPASAbs_S23_L001_R1_001.fastq.gz | EM23CoMCPASAbs_S23_L001_R2_001.fastq.gz |
| Bs_sa_mcpa_s3 | EM_24 | 16S rRNA in unplanted soil treated with syringic acid and MCPA | AMPLICON | METAGENOMIC | PCR | paired | ILLUMINA | Illumina MiSeq | 515F-806R primers | fastq | EM24CoMCPASAbs_S24_L001_R1_001.fastq.gz | EM24CoMCPASAbs_S24_L001_R2_001.fastq.gz |
| Rh_t1 | EM_25 | 16S rRNA in rhizospheric soil | AMPLICON | METAGENOMIC | PCR | paired | ILLUMINA | Illumina MiSeq | 515F-806R primers | fastq | EM25Zurh_S25_L001_R1_001.fastq.gz | EM25Zurh_S25_L001_R2_001.fastq.gz |
| Rh_t2 | EM_26 | 16S rRNA in rhizospheric soil | AMPLICON | METAGENOMIC | PCR | paired | ILLUMINA | Illumina MiSeq | 515F-806R primers | fastq | EM26Zurh_S26_L001_R1_001.fastq.gz | EM26Zurh_S26_L001_R2_001.fastq.gz |
| Rh_t3 | EM_27 | 16S rRNA in rhizospheric soil | AMPLICON | METAGENOMIC | PCR | paired | ILLUMINA | Illumina MiSeq | 515F-806R primers | fastq | EM27Zurh_S27_L001_R1_001.fastq.gz | EM27Zurh_S27_L001_R2_001.fastq.gz |
| Rh_s1 | EM_28 | 16S rRNA in rhizospheric soil | AMPLICON | METAGENOMIC | PCR | paired | ILLUMINA | Illumina MiSeq | 515F-806R primers | fastq | EM28Zurh_S28_L001_R1_001.fastq.gz | EM28Zurh_S28_L001_R2_001.fastq.gz |
| Rh_s2 | EM_29 | 16S rRNA in rhizospheric soil | AMPLICON | METAGENOMIC | PCR | paired | ILLUMINA | Illumina MiSeq | 515F-806R primers | fastq | EM29Zurh_S29_L001_R1_001.fastq.gz | EM29Zurh_S29_L001_R2_001.fastq.gz |
| Rh_s3 | EM_30 | 16S rRNA in rhizospheric soil | AMPLICON | METAGENOMIC | PCR | paired | ILLUMINA | Illumina MiSeq | 515F-806R primers | fastq | EM30Zurh_S30_L001_R1_001.fastq.gz | EM30Zurh_S30_L001_R2_001.fastq.gz |
| Rh_sa_t1 | EM_31 | 16S rRNA in rhizospheric soil treated with syringic acid | AMPLICON | METAGENOMIC | PCR | paired | ILLUMINA | Illumina MiSeq | 515F-806R primers | fastq | EM31ZuSArh_S31_L001_R1_001.fastq.gz | EM31ZuSArh_S31_L001_R2_001.fastq.gz |
| Rh_sa_t2 | EM_32 | 16S rRNA in rhizospheric soil treated with syringic acid | AMPLICON | METAGENOMIC | PCR | paired | ILLUMINA | Illumina MiSeq | 515F-806R primers | fastq | EM33ZuSArh_S32_L001_R1_001.fastq.gz | EM33ZuSArh_S32_L001_R2_001.fastq.gz |
| Rh_sa_t3 | EM_33 | 16S rRNA in rhizospheric soil treated with syringic acid | AMPLICON | METAGENOMIC | PCR | paired | ILLUMINA | Illumina MiSeq | 515F-806R primers | fastq | EM34ZuSArh_S33_L001_R1_001.fastq.gz | EM34ZuSArh_S33_L001_R2_001.fastq.gz |
| Rh_sa_s1 | EM_34 | 16S rRNA in rhizospheric soil treated with syringic acid | AMPLICON | METAGENOMIC | PCR | paired | ILLUMINA | Illumina MiSeq | 515F-806R primers | fastq | EM35ZuSArh_S34_L001_R1_001.fastq.gz | EM35ZuSArh_S34_L001_R2_001.fastq.gz |
| Rh_sa_s2 | EM_35 | 16S rRNA in rhizospheric soil treated with syringic acid | AMPLICON | METAGENOMIC | PCR | paired | ILLUMINA | Illumina MiSeq | 515F-806R primers | fastq | EM36ZuSArh_S35_L001_R1_001.fastq.gz | EM36ZuSArh_S35_L001_R2_001.fastq.gz |
| Rh_sa_s3 | EM_36 | 16S rRNA in rhizospheric soil treated with syringic acid | AMPLICON | METAGENOMIC | PCR | paired | ILLUMINA | Illumina MiSeq | 515F-806R primers | fastq | EM37ZuSArh_S36_L001_R1_001.fastq.gz | EM37ZuSArh_S36_L001_R2_001.fastq.gz |
| Rh_mcpa_t1 | EM_37 | 16S rRNA in rhizospheric soil treated with MCPA | AMPLICON | METAGENOMIC | PCR | paired | ILLUMINA | Illumina MiSeq | 515F-806R primers | fastq | EM38ZuMCPArh_S37_L001_R1_001.fastq.gz | EM38ZuMCPArh_S37_L001_R2_001.fastq.gz |
| Rh_mcpa_t2 | EM_38 | 16S rRNA in rhizospheric soil treated with MCPA | AMPLICON | METAGENOMIC | PCR | paired | ILLUMINA | Illumina MiSeq | 515F-806R primers | fastq | EM39ZuMCPArh_S38_L001_R1_001.fastq.gz | EM39ZuMCPArh_S38_L001_R2_001.fastq.gz |
| Rh_mcpa_t3 | EM_39 | 16S rRNA in rhizospheric soil treated with MCPA | AMPLICON | METAGENOMIC | PCR | paired | ILLUMINA | Illumina MiSeq | 515F-806R primers | fastq | EM40ZuMCPArh_S39_L001_R1_001.fastq.gz | EM40ZuMCPArh_S39_L001_R2_001.fastq.gz |
| Rh_mcpa_s1 | EM_40 | 16S rRNA in rhizospheric soil treated with MCPA | AMPLICON | METAGENOMIC | PCR | paired | ILLUMINA | Illumina MiSeq | 515F-806R primers | fastq | EM41ZuMCPArh_S40_L001_R1_001.fastq.gz | EM41ZuMCPArh_S40_L001_R2_001.fastq.gz |
| Rh_mcpa_s2 | EM_41 | 16S rRNA in rhizospheric soil treated with MCPA | AMPLICON | METAGENOMIC | PCR | paired | ILLUMINA | Illumina MiSeq | 515F-806R primers | fastq | EM42ZuMCPArh_S41_L001_R1_001.fastq.gz | EM42ZuMCPArh_S41_L001_R2_001.fastq.gz |
| Rh_mcpa_s3 | EM_42 | 16S rRNA in rhizospheric soil treated with MCPA | AMPLICON | METAGENOMIC | PCR | paired | ILLUMINA | Illumina MiSeq | 515F-806R primers | fastq | EM43ZuMCPArh_S42_L001_R1_001.fastq.gz | EM43ZuMCPArh_S42_L001_R2_001.fastq.gz |
| Rh_mcpa_sa_t1 | EM_43 | 16S rRNA in rhizospheric soil treated with syringic acid and MCPA | AMPLICON | METAGENOMIC | PCR | paired | ILLUMINA | Illumina MiSeq | 515F-806R primers | fastq | EM44ZuMCPASArh_S43_L001_R1_001.fastq.gz | EM44ZuMCPASArh_S43_L001_R2_001.fastq.gz |
| Rh_mcpa_sa_t2 | EM_44 | 16S rRNA in rhizospheric soil treated with syringic acid and MCPA | AMPLICON | METAGENOMIC | PCR | paired | ILLUMINA | Illumina MiSeq | 515F-806R primers | fastq | EM45ZuMCPASArh_S44_L001_R1_001.fastq.gz | EM45ZuMCPASArh_S44_L001_R2_001.fastq.gz |
| Rh_mcpa_sa_t3 | EM_45 | 16S rRNA in rhizospheric soil treated with syringic acid and MCPA | AMPLICON | METAGENOMIC | PCR | paired | ILLUMINA | Illumina MiSeq | 515F-806R primers | fastq | EM46ZuMCPASArh_S45_L001_R1_001.fastq.gz | EM46ZuMCPASArh_S45_L001_R2_001.fastq.gz |
| Rh_mcpa_sa_s1 | EM_46 | 16S rRNA in rhizospheric soil treated with syringic acid and MCPA | AMPLICON | METAGENOMIC | PCR | paired | ILLUMINA | Illumina MiSeq | 515F-806R primers | fastq | EM47ZuMCPASArh_S46_L001_R1_001.fastq.gz | EM47ZuMCPASArh_S46_L001_R2_001.fastq.gz |
| Rh_mcpa_sa_s2 | EM_47 | 16S rRNA in rhizospheric soil treated with syringic acid and MCPA | AMPLICON | METAGENOMIC | PCR | paired | ILLUMINA | Illumina MiSeq | 515F-806R primers | fastq | EM48ZuMCPASArh_S47_L001_R1_001.fastq.gz | EM48ZuMCPASArh_S47_L001_R2_001.fastq.gz |
| Rh_mcpa_sa_s3 | EM_48 | 16S rRNA in rhizospheric soil treated with syringic acid and MCPA | AMPLICON | METAGENOMIC | PCR | paired | ILLUMINA | Illumina MiSeq | 515F-806R primers | fastq | EM49ZuMCPASArh_S48_L001_R1_001.fastq.gz | EM49ZuMCPASArh_S48_L001_R2_001.fastq.gz |
| Ro_t1 | EM_49 | 16S rRNA in root | AMPLICON | METAGENOMIC | PCR | paired | ILLUMINA | Illumina MiSeq | 515F-806R primers | fastq | EM50Zuro_S49_L001_R1_001.fastq.gz | EM50Zuro_S49_L001_R2_001.fastq.gz |
| Ro_t2 | EM_50 | 16S rRNA in root | AMPLICON | METAGENOMIC | PCR | paired | ILLUMINA | Illumina MiSeq | 515F-806R primers | fastq | EM51Zuro_S50_L001_R1_001.fastq.gz | EM51Zuro_S50_L001_R2_001.fastq.gz |
| Ro_t3 | EM_51 | 16S rRNA in root | AMPLICON | METAGENOMIC | PCR | paired | ILLUMINA | Illumina MiSeq | 515F-806R primers | fastq | EM52Zuro_S51_L001_R1_001.fastq.gz | EM52Zuro_S51_L001_R2_001.fastq.gz |
| Ro_s1 | EM_52 | 16S rRNA in root | AMPLICON | METAGENOMIC | PCR | paired | ILLUMINA | Illumina MiSeq | 515F-806R primers | fastq | EM53Zuro_S52_L001_R1_001.fastq.gz | EM53Zuro_S52_L001_R2_001.fastq.gz |
| Ro_s2 | EM_53 | 16S rRNA in root | AMPLICON | METAGENOMIC | PCR | paired | ILLUMINA | Illumina MiSeq | 515F-806R primers | fastq | EM54Zuro_S53_L001_R1_001.fastq.gz | EM54Zuro_S53_L001_R2_001.fastq.gz |
| Ro_s3 | EM_54 | 16S rRNA in root | AMPLICON | METAGENOMIC | PCR | paired | ILLUMINA | Illumina MiSeq | 515F-806R primers | fastq | EM55Zuro_S54_L001_R1_001.fastq.gz | EM55Zuro_S54_L001_R2_001.fastq.gz |
| Ro_sa_t1 | EM_55 | 16S rRNA in root treated with syringic acid | AMPLICON | METAGENOMIC | PCR | paired | ILLUMINA | Illumina MiSeq | 515F-806R primers | fastq | EM56ZuSAro_S55_L001_R1_001.fastq.gz | EM56ZuSAro_S55_L001_R2_001.fastq.gz |
| Ro_sa_t2 | EM_56 | 16S rRNA in root treated with syringic acid | AMPLICON | METAGENOMIC | PCR | paired | ILLUMINA | Illumina MiSeq | 515F-806R primers | fastq | EM57ZuSAro_S56_L001_R1_001.fastq.gz | EM57ZuSAro_S56_L001_R2_001.fastq.gz |
| Ro_sa_t3 | EM_57 | 16S rRNA in root treated with syringic acid | AMPLICON | METAGENOMIC | PCR | paired | ILLUMINA | Illumina MiSeq | 515F-806R primers | fastq | EM58ZuSAro_S57_L001_R1_001.fastq.gz | EM58ZuSAro_S57_L001_R2_001.fastq.gz |
| Ro_sa_s1 | EM_58 | 16S rRNA in root treated with syringic acid | AMPLICON | METAGENOMIC | PCR | paired | ILLUMINA | Illumina MiSeq | 515F-806R primers | fastq | EM59ZuSAro_S58_L001_R1_001.fastq.gz | EM59ZuSAro_S58_L001_R2_001.fastq.gz |
| Ro_sa_s2 | EM_59 | 16S rRNA in root treated with syringic acid | AMPLICON | METAGENOMIC | PCR | paired | ILLUMINA | Illumina MiSeq | 515F-806R primers | fastq | EM60ZuSAro_S59_L001_R1_001.fastq.gz | EM60ZuSAro_S59_L001_R2_001.fastq.gz |
| Ro_sa_s3 | EM_60 | 16S rRNA in root treated with syringic acid | AMPLICON | METAGENOMIC | PCR | paired | ILLUMINA | Illumina MiSeq | 515F-806R primers | fastq | EM61ZuSAro_S60_L001_R1_001.fastq.gz | EM61ZuSAro_S60_L001_R2_001.fastq.gz |
| Ro_mcpa_t1 | EM_61 | 16S rRNA in root treated with MCPA | AMPLICON | METAGENOMIC | PCR | paired | ILLUMINA | Illumina MiSeq | 515F-806R primers | fastq | EM62ZuMCPAro_S61_L001_R1_001.fastq.gz | EM62ZuMCPAro_S61_L001_R2_001.fastq.gz |
| Ro_mcpa_t2 | EM_62 | 16S rRNA in root treated with MCPA | AMPLICON | METAGENOMIC | PCR | paired | ILLUMINA | Illumina MiSeq | 515F-806R primers | fastq | EM63ZuMCPAro_S62_L001_R1_001.fastq.gz | EM63ZuMCPAro_S62_L001_R2_001.fastq.gz |
| Ro_mcpa_t3 | EM_63 | 16S rRNA in root treated with MCPA | AMPLICON | METAGENOMIC | PCR | paired | ILLUMINA | Illumina MiSeq | 515F-806R primers | fastq | EM64ZuMCPAro_S63_L001_R1_001.fastq.gz | EM64ZuMCPAro_S63_L001_R2_001.fastq.gz |
| Ro_mcpa_s1 | EM_64 | 16S rRNA in root treated with MCPA | AMPLICON | METAGENOMIC | PCR | paired | ILLUMINA | Illumina MiSeq | 515F-806R primers | fastq | EM65ZuMCPAro_S64_L001_R1_001.fastq.gz | EM65ZuMCPAro_S64_L001_R2_001.fastq.gz |
| Ro_mcpa_s2 | EM_65 | 16S rRNA in root treated with MCPA | AMPLICON | METAGENOMIC | PCR | paired | ILLUMINA | Illumina MiSeq | 515F-806R primers | fastq | EM66ZuMCPAro_S65_L001_R1_001.fastq.gz | EM66ZuMCPAro_S65_L001_R2_001.fastq.gz |
| Ro_mcpa_s3 | EM_66 | 16S rRNA in root treated with MCPA | AMPLICON | METAGENOMIC | PCR | paired | ILLUMINA | Illumina MiSeq | 515F-806R primers | fastq | EM67ZuMCPAro_S66_L001_R1_001.fastq.gz | EM67ZuMCPAro_S66_L001_R2_001.fastq.gz |
| Ro_sa_mcpa_t1 | EM_67 | 16S rRNA in root treated with syringic acid and MCPA | AMPLICON | METAGENOMIC | PCR | paired | ILLUMINA | Illumina MiSeq | 515F-806R primers | fastq | EM68ZuMCPASAro_S67_L001_R1_001.fastq.gz | EM68ZuMCPASAro_S67_L001_R2_001.fastq.gz |
| Ro_sa_mcpa_t2 | EM_68 | 16S rRNA in root treated with syringic acid and MCPA | AMPLICON | METAGENOMIC | PCR | paired | ILLUMINA | Illumina MiSeq | 515F-806R primers | fastq | EM69ZuMCPASAro_S68_L001_R1_001.fastq.gz | EM69ZuMCPASAro_S68_L001_R2_001.fastq.gz |
| Ro_sa_mcpa_t3 | EM_69 | 16S rRNA in root treated with syringic acid and MCPA | AMPLICON | METAGENOMIC | PCR | paired | ILLUMINA | Illumina MiSeq | 515F-806R primers | fastq | EM70ZuMCPASAro_S69_L001_R1_001.fastq.gz | EM70ZuMCPASAro_S69_L001_R2_001.fastq.gz |
| Ro_sa_mcpa_s1 | EM_70 | 16S rRNA in root treated with syringic acid and MCPA | AMPLICON | METAGENOMIC | PCR | paired | ILLUMINA | Illumina MiSeq | 515F-806R primers | fastq | EM71ZuMCPASAro_S71_L001_R1_001.fastq.gz | EM71ZuMCPASAro_S71_L001_R2_001.fastq.gz |
| Ro_sa_mcpa_s2 | EM_71 | 16S rRNA in root treated with syringic acid and MCPA | AMPLICON | METAGENOMIC | PCR | paired | ILLUMINA | Illumina MiSeq | 515F-806R primers | fastq | EM72ZuMCPASAro_S70_L001_R1_001.fastq.gz | EM72ZuMCPASAro_S70_L001_R2_001.fastq.gz |
| Ro_sa_mcpa_s3 | EM_72 | 16S rRNA in root treated with syringic acid and MCPA | AMPLICON | METAGENOMIC | PCR | paired | ILLUMINA | Illumina MiSeq | 515F-806R primers | fastq | EM73ZuMCPASAro_S72_L001_R1_001.fastq.gz | EM73ZuMCPASAro_S72_L001_R2_001.fastq.gz |
| Le_t1 | EM_73 | 16S rRNA in leaf | AMPLICON | METAGENOMIC | PCR | paired | ILLUMINA | Illumina MiSeq | 515F-806R primers | fastq | EM74Zule_S73_L001_R1_001.fastq.gz | EM74Zule_S73_L001_R2_001.fastq.gz |
| Le_t2 | EM_74 | 16S rRNA in leaf | AMPLICON | METAGENOMIC | PCR | paired | ILLUMINA | Illumina MiSeq | 515F-806R primers | fastq | EM75Zule_S74_L001_R1_001.fastq.gz | EM75Zule_S74_L001_R2_001.fastq.gz |
| Le_t3 | EM_75 | 16S rRNA in leaf | AMPLICON | METAGENOMIC | PCR | paired | ILLUMINA | Illumina MiSeq | 515F-806R primers | fastq | EM76Zule_S75_L001_R1_001.fastq.gz | EM76Zule_S75_L001_R2_001.fastq.gz |
| Le_s1 | EM_76 | 16S rRNA in leaf | AMPLICON | METAGENOMIC | PCR | paired | ILLUMINA | Illumina MiSeq | 515F-806R primers | fastq | EM77Zule_S76_L001_R1_001.fastq.gz | EM77Zule_S76_L001_R2_001.fastq.gz |
| Le_s2 | EM_77 | 16S rRNA in leaf | AMPLICON | METAGENOMIC | PCR | paired | ILLUMINA | Illumina MiSeq | 515F-806R primers | fastq | EM78Zule_S77_L001_R1_001.fastq.gz | EM78Zule_S77_L001_R2_001.fastq.gz |
| Le_s3 | EM_78 | 16S rRNA in leaf | AMPLICON | METAGENOMIC | PCR | paired | ILLUMINA | Illumina MiSeq | 515F-806R primers | fastq | EM79Zule_S78_L001_R1_001.fastq.gz | EM79Zule_S78_L001_R2_001.fastq.gz |
| Le_sa_t1 | EM_79 | 16S rRNA in leaf treated with syringic acid | AMPLICON | METAGENOMIC | PCR | paired | ILLUMINA | Illumina MiSeq | 515F-806R primers | fastq | EM80ZuSAle_S79_L001_R1_001.fastq.gz | EM80ZuSAle_S79_L001_R2_001.fastq.gz |
| Le_sa_t2 | EM_80 | 16S rRNA in leaf treated with syringic acid | AMPLICON | METAGENOMIC | PCR | paired | ILLUMINA | Illumina MiSeq | 515F-806R primers | fastq | EM81ZuSAle_S80_L001_R1_001.fastq.gz | EM81ZuSAle_S80_L001_R2_001.fastq.gz |
| Le_sa_t3 | EM_81 | 16S rRNA in leaf treated with syringic acid | AMPLICON | METAGENOMIC | PCR | paired | ILLUMINA | Illumina MiSeq | 515F-806R primers | fastq | EM82ZuSAle_S81_L001_R1_001.fastq.gz | EM82ZuSAle_S81_L001_R2_001.fastq.gz |
| Le_sa_s1 | EM_82 | 16S rRNA in leaf treated with syringic acid | AMPLICON | METAGENOMIC | PCR | paired | ILLUMINA | Illumina MiSeq | 515F-806R primers | fastq | EM83ZuSAle_S82_L001_R1_001.fastq.gz | EM83ZuSAle_S82_L001_R2_001.fastq.gz |
| Le_sa_s2 | EM_83 | 16S rRNA in leaf treated with syringic acid | AMPLICON | METAGENOMIC | PCR | paired | ILLUMINA | Illumina MiSeq | 515F-806R primers | fastq | EM84ZuSAle_S83_L001_R1_001.fastq.gz | EM84ZuSAle_S83_L001_R2_001.fastq.gz |
| Le_sa_s3 | EM_84 | 16S rRNA in leaf treated with syringic acid | AMPLICON | METAGENOMIC | PCR | paired | ILLUMINA | Illumina MiSeq | 515F-806R primers | fastq | EM85ZuSAle_S84_L001_R1_001.fastq.gz | EM85ZuSAle_S84_L001_R2_001.fastq.gz |
| Le_mcpa_t1 | EM_85 | 16S rRNA in leaf treated with MCPA | AMPLICON | METAGENOMIC | PCR | paired | ILLUMINA | Illumina MiSeq | 515F-806R primers | fastq | EM86ZuMCPAle_S85_L001_R1_001.fastq.gz | EM86ZuMCPAle_S85_L001_R2_001.fastq.gz |
| Le_mcpa_t2 | EM_86 | 16S rRNA in leaf treated with MCPA | AMPLICON | METAGENOMIC | PCR | paired | ILLUMINA | Illumina MiSeq | 515F-806R primers | fastq | EM87ZuMCPAle_S86_L001_R1_001.fastq.gz | EM87ZuMCPAle_S86_L001_R2_001.fastq.gz |
| Le_mcpa_t3 | EM_87 | 16S rRNA in leaf treated with MCPA | AMPLICON | METAGENOMIC | PCR | paired | ILLUMINA | Illumina MiSeq | 515F-806R primers | fastq | EM88ZuMCPAle_S87_L001_R1_001.fastq.gz | EM88ZuMCPAle_S87_L001_R2_001.fastq.gz |
| Le_mcpa_s1 | EM_88 | 16S rRNA in leaf treated with MCPA | AMPLICON | METAGENOMIC | PCR | paired | ILLUMINA | Illumina MiSeq | 515F-806R primers | fastq | EM89ZuMCPAle_S88_L001_R1_001.fastq.gz | EM89ZuMCPAle_S88_L001_R2_001.fastq.gz |
| Le_mcpa_s2 | EM_89 | 16S rRNA in leaf treated with MCPA | AMPLICON | METAGENOMIC | PCR | paired | ILLUMINA | Illumina MiSeq | 515F-806R primers | fastq | EM90ZuMCPAle_S89_L001_R1_001.fastq.gz | EM90ZuMCPAle_S89_L001_R2_001.fastq.gz |
| Le_mcpa_s3 | EM_90 | 16S rRNA in leaf treated with MCPA | AMPLICON | METAGENOMIC | PCR | paired | ILLUMINA | Illumina MiSeq | 515F-806R primers | fastq | EM91ZuMCPAle_S90_L001_R1_001.fastq.gz | EM91ZuMCPAle_S90_L001_R2_001.fastq.gz |
| Le_mcpa_sa_t1 | EM_91 | 16S rRNA in leaf treated with syringic acid and MCPA | AMPLICON | METAGENOMIC | PCR | paired | ILLUMINA | Illumina MiSeq | 515F-806R primers | fastq | EM92ZuMCPASAle_S91_L001_R1_001.fastq.gz | EM92ZuMCPASAle_S91_L001_R2_001.fastq.gz |
| Le_mcpa_sa_t2 | EM_92 | 16S rRNA in leaf treated with syringic acid and MCPA | AMPLICON | METAGENOMIC | PCR | paired | ILLUMINA | Illumina MiSeq | 515F-806R primers | fastq | EM93ZuMCPASAle_S92_L001_R1_001.fastq.gz | EM93ZuMCPASAle_S92_L001_R2_001.fastq.gz |
| Le_mcpa_sa_t3 | EM_93 | 16S rRNA in leaf treated with syringic acid and MCPA | AMPLICON | METAGENOMIC | PCR | paired | ILLUMINA | Illumina MiSeq | 515F-806R primers | fastq | EM94ZuMCPASAle_S93_L001_R1_001.fastq.gz | EM94ZuMCPASAle_S93_L001_R2_001.fastq.gz |
| Le_mcpa_sa_s1 | EM_94 | 16S rRNA in leaf treated with syringic acid and MCPA | AMPLICON | METAGENOMIC | PCR | paired | ILLUMINA | Illumina MiSeq | 515F-806R primers | fastq | EM95ZuMCPASAle_S94_L001_R1_001.fastq.gz | EM95ZuMCPASAle_S94_L001_R2_001.fastq.gz |
| Le_mcpa_sa_s2 | EM_95 | 16S rRNA in leaf treated with syringic acid and MCPA | AMPLICON | METAGENOMIC | PCR | paired | ILLUMINA | Illumina MiSeq | 515F-806R primers | fastq | EM96ZuMCPASAle_S95_L001_R1_001.fastq.gz | EM96ZuMCPASAle_S95_L001_R2_001.fastq.gz |
| Le_mcpa_sa_s3 | EM_96 | 16S rRNA in leaf treated with syringic acid and MCPA | AMPLICON | METAGENOMIC | PCR | paired | ILLUMINA | Illumina MiSeq | 515F-806R primers | fastq | EM97ZuMCPASAle_S96_L001_R1_001.fastq.gz | EM97ZuMCPASAle_S96_L001_R2_001.fastq.gz |
| EMDNABlank | EMDNABlank | blank | AMPLICON | METAGENOMIC | PCR | paired | ILLUMINA | Illumina MiSeq | 515F-806R primers | fastq | EMDNABlank_S97_L001_R1_001.fastq.gz | EMDNABlank_S97_L001_R2_001.fastq.gz |
| MockB | MockB | mock community | AMPLICON | METAGENOMIC | PCR | paired | ILLUMINA | Illumina MiSeq | 515F-806R primers | fastq | MockB_S98_L001_R1_001.fastq.gz | MockB_S98_L001_R2_001.fastq.gz |
